# Intensive grazing alters the diversity, composition and structure of plant-pollinator interaction networks in Central European grasslands

**DOI:** 10.1371/journal.pone.0263576

**Published:** 2022-03-11

**Authors:** Demetra Rakosy, Elena Motivans, Valentin Ştefan, Arkadiusz Nowak, Sebastian Świerszcz, Reinart Feldmann, Elisabeth Kühn, Costanza Geppert, Neeraja Venkataraman, Anna Sobieraj-Betlińska, Anita Grossmann, Wiktoria Rojek, Katarzyna Pochrząst, Magdalena Cielniak, Anika Kirstin Gathof, Kevin Baumann, Tiffany Marie Knight

**Affiliations:** 1 Department for Community Ecology, Helmholtz Centre for Environmental Research–UFZ, Leipzig, Germany; 2 German Centre for Integrative Biodiversity Research (iDiv) Halle-Jena-Leipzig, Leipzig, Germany; 3 Institute of Biology, Martin Luther University Halle-Wittenberg, Halle (Saale), Germany; 4 Center for Biological Diversity Conservation, Polish Academy of Sciences, Botanical Garden, Warsaw, Poland; 5 Institute of Biology, University of Opole, Opole, Poland; 6 Polish Academy of Sciences, The Franciszek Górski Institute of Plant Physiology, Opole, Poland; 7 Helmholtz Centre for Environmental Research–UFZ, Leipzig, Germany; 8 Department of Agronomy, Food, Natural Resources, Animals and Environment, University of Padova School of Agricultural Sciences and Veterinary Medicine, Padova, Italy; 9 Department of Environmental Biology, Faculty of Biological Sciences, Kazimierz Wielki University, Bydgoszcz, Poland; 10 Department of Ecology, Chair of Ecosystem Sciences/Plant Ecology, Technical University Berlin, Berlin, Germany; 11 Institute of Environmental Sciences, Jagiellonian University, Krakow, Poland; 12 Faculty of Natural Sciences and Technology, University of Opole, Opole, Poland; 13 IFZ–Department for Animal Ecology, Justus Liebig University, Gießen, Germany; University of the Balearic Islands, SPAIN

## Abstract

Complex socio-economic, political and demographic factors have driven the increased conversion of Europe’s semi-natural grasslands to intensive pastures. This trend is particularly strong in some of the most biodiverse regions of the continent, such as Central and Eastern Europe. Intensive grazing is known to decrease species diversity and alter the composition of plant and insect communities. Comparatively little is known, however, about how intensive grazing influences plant functional traits related to pollination and the structure of plant-pollinator interactions. In traditional hay meadows and intensive pastures in Central Europe, we contrasted the taxonomic and functional group diversity and composition, the structure of plant-pollinator interactions and the roles of individual species in networks. We found mostly lower taxonomic and functional diversity of plants and insects in intensive pastures, as well as strong compositional differences among the two grassland management types. Intensive pastures were dominated by a single plant with a specialized flower structure that is only accessible to a few pollinator groups. As a result, intensive pastures have lower diversity and specificity of interactions, higher amount of resource overlap, more uniform interaction strength and lower network modularity. These findings stand in contrast to studies in which plants with more generalized flower traits dominated pastures. Our results thus highlight the importance of the functional traits of dominant species in mediating the consequences of intensive pasture management on plant-pollinator networks. These findings could further contribute to strategies aimed at mitigating the impact of intensive grazing on plant and pollinator communities.

## Introduction

Europe’s diverse semi-natural grasslands are being reshaped by intensive anthropogenic change [1, 2]. As a consequence, almost all European countries report a decline in the area covered by semi-natural grasslands and an associated loss of biodiversity [1, 3, 4]. This has led to increasing concern that European grasslands will not be able to maintain essential ecosystem services, such as pollination [5, 6]. Pollination services occur in a community context, where plant-pollinator encounters are embedded within an interaction network [7–10]. Plant-pollinator interaction networks quantify the frequency of pairwise interactions between plants and pollinators [11], their structure being shaped by the diversity, abundance and composition of both plants and pollinators [11]. Quantifying how plant-pollinator networks are altered by anthropogenic change is key to understanding and predicting shifts in interaction structure and their consequences for grassland ecosystem services.

Among semi-natural grasslands, intensively managed, permanent pastures and traditionally managed hay meadows (this is known as extensive management) represent two opposite endpoints of disturbance and nutrient input gradients [12]. Traditionally managed hay meadows are subjected to low disturbance and nutrient input regimes, as plant biomass is removed only once or twice a year and fertilizer input is minimal [13, 14]. Intensive, permanent pastures are, in contrast, characterized by prolonged grazing and high animal stocking rate, leading to constant disturbance and high nutrient input throughout the year [12]. The conversion of traditional hay meadows to intensive pastures is driven by a complex array of socio-economic, political and demographic factors [15, 16], and is particularly common in Central and Eastern European countries [16–18]. These intensive pastures are defined by low species diversity and are often dominated by grazing-, trampling- and nutrient-tolerant plant species [18–21]. Such disturbance-induced shifts in plant species diversity and composition could have consequences for higher trophic levels, such as pollinating insects [22–24]. This is because plant diversity is usually correlated with pollinator diversity, a correlation hypothesized to be mediated by floral resource availability and accessibility [25–27].

The diversity and composition of pollinators should thus depend on plant species diversity, plant functional composition and functional trait matching between plants and pollinators. Hay meadows with diverse plant communities will harbour a broad range of flowers that provide resources that are accessible to a broad spectrum of pollinators, including specialists [28, 29]. Intensive pastures will in turn contain only a few plant species with vegetative traits that tolerate intensive management regimes [19, 30]. Depending on their geographic location, environmental conditions and the type and stocking rate of herbivores, pastures could be dominated by grazing-tolerant plant species with either generalist or specialist floral functional traits. A recent review has highlighted the lack of data on the effect of grazing on floral traits, with the available research indicating that generalist flowers could be favoured [31]. However, some studied reveal that Fabaceae, which have quite specialized flowers, are often dominant in intensive pastures throughout Europe (i.e. [32–34]). Differences in the dominance of particular floral traits might explain why the effect of grazing on pollinators and plant-pollinator interactions is variable across studies [30, 35–38].

If plants with generalist flower traits (i.e. that provide accessible resources to a broader range of pollinators) dominate in intensive pastures, then intensive grazing should have only a minor effect on pollinator diversity, community composition and thus on network architecture [36, 39]. Such was the case in Brazil, where the dominance of Asteraceae species in intensive pastures resulted in only small changes in network structure compared to less intensely managed sites [36]. Alternatively, if plants with specialized floral traits dominate in intensive pastures, significant shifts in network structure should occur including: a decrease in the frequency of unique interactions and thus interaction diversity as a consequence of the loss of diversity of pollinators with traits which do not match the dominant plant species; a decrease of interaction evenness as a consequence of a skewed distribution of interactions towards the dominant plant species; an increase in network-level specialization and niche overlap, as all pollinators primarily utilize the dominant plant species; the loss of entire groups of interacting and functionally matching species (i.e., modules); and an increased role of the dominant plant species, as well as its interaction partners, in the network.

However, the impact of the dominance of plant species with specialized floral traits on pollinator diversity and composition and on plant-pollinator networks has so far received only very limited attention. In the present study we thus aim to assess the effects of intensive grazing on the diversity, composition and structure of interactions between plant and pollinator communities. We thereby explicitly consider the changes in floral functional traits and their importance in determining the role of particular species within plant-pollinator networks and in network responses to intensive grazing. The following questions are specifically addressed: i) Do plant and pollinator communities have lower taxonomic and functional diversity in intensive pastures compared to hay meadows? ii) Does intensive grazing shift plant and insect communities towards the dominance of a few species or groups? iii) Does intensive grazing shift network structure and the roles species play within networks?

## Materials and methods

### Study sites

The study was conducted in the Opawskie Mountains, at the border between Poland and the Czech Republic (S1 Table). We selected five semi-natural grassland sites found at the two extreme ends of a disturbance and nutrient input gradient. From the five sites, two were unimproved (i.e. no additional sowing of plant species for fodder), permanent, intensively grazed cow pastures (high disturbance, high nutrient input) and three were extensive hay meadows, mown only once or twice a year and not fertilized (low disturbance, low nutrient input). All sites were imbedded in a similar landscape matrix and occurred on similar mesic, acidic soils and within the same altitudinal zone. Vegetation in intensive pastures corresponded to *Lolio perennis-Cynosuretum cristati* phytosociological association(*Cynosurion cristati* alliance), whereas that of hay meadows corresponded to *Poo-Trisetetum flavescentis* phytoassociation (*Arrhenatherion eatioris* alliance) (see also [33]); which are linked to low and high land use intensity (respectively). In fact, additional analyses confirmed that vegetation in pastures was dominated by species tolerant to grazing, trampling and increased nutrient inputs and could thus be classified as intensively managed (S1 Appendix and S2 Table). Field work was conducted with the consent from local land owners and in accordance with applicable law (as no threatened species were collected, no collection permits were required).

### Species sampling

Sampling took place during peak flowering in the middle of June 2018. We chose this point in time because it represents the period when most flowering plants are in bloom, and in the case of the hay meadows it reflects the maximum development of the vegetation before mowing (see also [38]). Within each of the five sites, we established 10 transects (with the exception of one pasture for which we could only place 6 transects), each measuring 30x2m. Transects were placed with a minimum distance of 30m between them, and towards the nearest field margins. In each transect, we visually estimated the percent cover of flowers/inflorescences of each plant species (i.e. [40]). We used the standardized transect walks to also quantify pollinator species and plant-pollinator interactions. One collector walked each transect for an active sampling period of 15 min. We thereby sampled over 3 days in total (for 300 min in pastures and 450 min in hay meadows), with two days in which a pasture and a hay meadow site were sampled in parallel, and a day in which the remaining hay meadow was sampled.

All Hymenoptera, Diptera and Lepidoptera that contacted the reproductive structures of the flowers were treated as potential pollinators and collected using sweeping nets (we hereafter refer to these groups as pollinators for simplicity); easily identifiable species were identified on site and released, while all other individuals were collected and frozen for further processing. All plant species were identified to species level. Among pollinators, 85.5% of all individuals were identified to species level and 99.5% to genus level (see species lists in S2 and S3 Tables).

### Sampling completeness

Most sampling methods are incomplete; as such no study is likely to capture the entire complexity of biological communities [41, 42]. In order to ensure that our sampling has captured a comparable completeness of the community diversity across all sites and management types, we evaluated the sampling completeness of flowering plants, their pollinators and unique plant-pollinator interactions [42]. We used the bias-corrected Chao2 estimator of asymptotic richness for quantifying the percentage of asymptotic richness detected by the observed richness [43]. Calculations were performed using the “specpool” function in the package vegan in R [44]. Sampling completeness among the two grassland management types was compared using the unpaired two-sample Wilcoxon test in R.

### Functional trait classifications

We categorized all flowering species based on their flower structure and the accessibility and type of reward using the BiolFlor database (*www.biolflor.de*, [45]) and a slightly modified version of the classification of [46]. We thus grouped plant species into nine functional groups: i) flower heads (nectar hidden at the base of individual florets); ii) disk flowers with open nectar; iii) disk flowers with hidden nectar; iv) lip flowers (nectar hidden at the base of the flower); v) pollen flowers (pollen as the only, openly available reward); vi) bell shaped flowers (nectar more or less hidden within the flowers); vii) flag flowers (nectar hidden at the base of the flowers); viii) stalk disk flowers (nectar hidden very deep within the flowers); ix) funnel flowers (nectar hidden at the base of the flowers) (S1 Fig).

For pollinators, the length of the proboscis together with innate visual and olfactory preferences for particular flower types, are important in influencing a pollinator’s flower choice. Information about the mean proboscis length of each recorded pollinator species was compiled from a broad range of sources (i.e. [47–50]). For the Diptera, only the Syrphidae could be included as information on other families is missing. As the data on the length of the proboscis stems from different sources and has been measured in different ways, we grouped tongue lengths into three categories: (i) short (1–5 mm); (ii) medium (5.5–9.5 mm); (iii) long (> 10 mm). In order to roughly account for the innate preferences of pollinator groups (assuming that particular orders differ in their flower preferences as suggested by the floral syndrome hypothesis, [28]), we differentiated among the three tongue length categories within Hymenoptera, Lepidoptera and Diptera (only Syrphidae). Functional groupings for all plant and pollinator species can be found in S2 and S3 Tables.

### Data analysis

Unless otherwise specified, analyses were performed by pooling transects within sites and sites within management types in order to compare the two grassland management types. We also performed all analyses by pooling transects within sites in order to assess site-specific variation within management types; however, as site-specific results were consistent with the overall analyses we only present overall analyses performed at management type level.

#### a) Comparison of species and functional diversity

While sampling completeness was not significantly different among the two management types (see Results), sampling effort (e.g. the number of transects sampled per management type) was. We thus rarefied and extrapolated species and functional richness and diversity of plants and their pollinators by transect [51, 52]. Plant cover and insect frequencies were converted to incidences (presence or absence) before incidence-based methods were applied. In order to obtain an estimate of richness and diversity from a functional perspective (referred to as functional richness and diversity throughout the manuscript) we substituted species by their functional groups. We used Hill numbers to quantify species and functional richness (q = 0), Shannon diversity (q = 1) and Simpson diversity (q = 2). Hill numbers are an effective way to compare diversity of species rich communities and allow a systematic assessment of the effect of dominant and rare species [53]. All diversity measures were performed using the function “iNext” in the package iNEXT in R [51]. Interpolated rarefaction was based on random sampling of transects, with extrapolation performed to twice the number of transects sampled [51]. The comparison of richness and diversity of pastures in relation to hay meadows was performed by constructing 95% confidence intervals, with non-overlapping 95% confidence intervals at the same number of sampling units indicating significant differences among management types [52, 54].

#### b) Assessing changes in community composition: Patterns of taxonomic and functional dissimilarity and dominance

In order to assess whether plant and insect species and functional groups of pastures represent a subset of the species and functional groups found in hay meadows, or whether they have been replaced by species more adapted to intensive management, we quantified the amount of turnover and nestedness in plant and insect communities between management types using the incidence-based Sørensen dissimilarity index [55, 56]. Overall dissimilarity partitioning among grassland management types was performed using the function “beta.multi” in the “betapart” package in R [57].

Dominance patterns of plant and pollinator species and functional groups were compared by performing a non-parametric multivariate ANOVA-type test in the R package “npmv” [58]. Function “nonpartest” with 1000 permutations was used to assess the overall distribution of the relative abundances of plant (measured as relative cover) and pollinator species (measured as relative frequency) in each management types. We thereby focused only on the 10 most abundant plant and pollinator species within each grassland management type. In order to determine between which species significant difference occurred we relied on the function “ssnonpartest”, using F-approximations for ANOVA Type statistics, from the same package. This function is an equivalent to follow-up ANOVA tests usually performed in parametric procedures. A similar approach was used for comparing the dominance patterns of plant and pollinator functional groups (all functional groups were included; for this analysis we replaced species with their functional group and recalculated relative cover for each functional group per management type).

#### c) Plant-pollinator network structure and changes in species roles

We constructed visitation networks for hay meadows and intensive pastures based on interaction frequency between plants and pollinators using the ‘plotweb’ function in the bipartite R package [59]. For visualization purposes, we summarized insect species into genera, however all analyses were performed at the species level. All graphical outputs were created in R vers. 1.2.5033, with visual properties (colour, lines, labelling) being further adapted using Corel Draw (vers. 20.0.0.633).

Network analyses relied on weighted, quantitative metrics chosen to provide insights into the way that changes in the diversity, composition and dominance patterns of species can alter the structure and stability of plant-pollinator networks. Specifically, we assessed metrics which account for:

- Changes in diversity and evenness of interactions–*Shannon diversity of interactions and interaction evenness*.
- Changes in the specificity of interactions–*H2* and *niche overlap*. *H2* is a measure of specialization and reflects link complementary across all species in the network [60]. The value of the metric can range between 0 (no specialization) and 1 (high dependency of each species in the network to a narrow range of partners). While *H2* reflects the number of interaction partners, *niche overlap* reveals the extent to which partners are shared among either plants or pollinators.
- Changes in the degree of compartmentalization of interactions (*modularity)*. This metric reflects changes in diversity, evenness of interactions and their specificity, providing insights into the stability of networks [61–63]. A modular network is considered to be more stable, as it consists of groups of species that interact more frequently with each other than with species belonging to other groups. The loss of particular species or even a module is thus less likely to have a cascading effect throughout the entire network [64].

Comparisons between intensive pastures and traditionally managed hay meadows were performed by rarefying all network-level metrics, except modularity, for number of interactions sampled per management type and iterated 100 times with 95% confidence intervals using the function “boot_networklevel” in the package “bootstrapnet” [65]. This function randomly samples interactions without replacement to build an interaction-based rarefaction curve for each metric [65]. The resulting rarefaction curves represent the mean of the 100 iterations and end in the value generated by the “networklevel” function in bipartite. Non-overlapping 95% confidence intervals at an equal number of interactions show significant differences among the network indices [65]. This approach allows metrics to be compared between management types at a standard number of interactions sampled [65]. In order to assess the level of modularity and the number of modules for networks of each management type we used the “metaComputeModule” function in bipartite, using the DIRT_LPA_wb_plus algorithm (default settings) [64]. Functional traits of both plants and pollinators were visualized to aid with the interpretation of the observed modules (i.e. [66]).

Among all plant species common to both hay meadows and pastures, *T*. *repens* experienced the largest shift in dominance, becoming the single most dominant species in pastures (see Results). Consequently, we chose this species and its most frequent pollinators in order to assess whether and how shifts in dominance patterns of species led to changes to their roles across management types. Changes in species roles within networks were measured by calculating the following species-level metrics:

- Species strength: quantifies the importance of a species among all its partners, based on the sum of dependencies of each species [67, 68]. Species with high values of this metric are considered keystone species within networks, as it shows that a higher number of interaction partners are dependent upon them [36, 69].
- Partner diversity: reflects the diversity of interaction partners of each species and is calculated as the weighted mean Shannon diversity index for all interactions of that particular species. High values indicate an even spread of interactions across partners, while low values indicate that the species interacts preferentially with one or a few partners [59].
- Species specialization (d’): indicates the degree of specialization of a species, by measuring how much a specie’s resource use deviates from opportunism. Its values range from 0 (no specialization) to 1 (complete specialization), with low values reflecting either interactions with multiple species or with a single dominant species and high values reflecting either interactions with just one or a few species or a several very rare species [70].

For all metrics we build an interaction-based rarefaction curve using the “bootstrapnet” package, function “specieslevel”, as described for network-level metrics above [65].

## Results

We recorded a total of 67 flowering plant species belonging to 23 families, 108 pollinator species belonging to 24 families (Hymenoptera: 19 species, 4 families; Diptera: 68 species, 14 families; Lepidoptera: 21 species, 7 families) and a total of 1767 unique interactions between plant and pollinator species (S2 and S3 Tables).

### Sampling completeness

We found no significant differences in the sampling completeness of plants (W = 4; p = 0.773), pollinators (W = 3; p = 1) and interactions (W = 3; p = 1) among intensive pastures and hay meadows. Sampling completeness in intensive pastures thereby reached a median value of 65.6 (IQR = 5.58) for plants, 54.6(IQR = 6.18) for pollinators and 40.8 (IQR = 2.49) for interactions. In comparison, sampling completeness in hay meadows reached median value of 74.8 (interquartile range = 16) for plants, 55.9 (IQR = 14) for pollinators and 40.3 (IQR = 7.23) for interactions.

### Species and functional diversity

**Fig 1:**
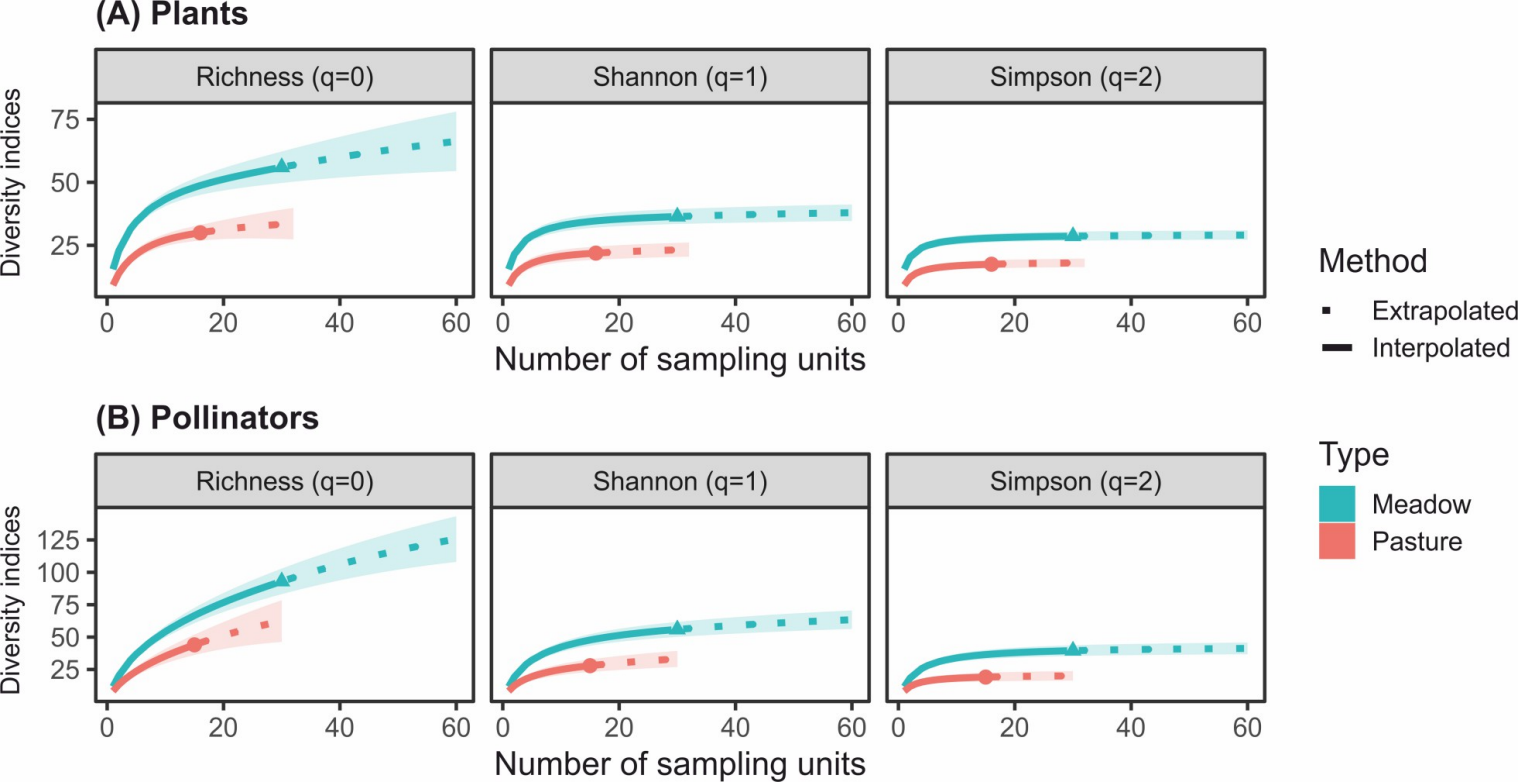
Differences in species richness and diversity between extensive pastures and traditional hay meadows. Sampling-unit (i.e. transects) based interpolation (continuous lines) and extrapolation (dashed lines, up to double the number of sampling units) of (A) plant and (B) pollinator species richness and diversity using Hill numbers. Intensive pastures had significantly lower plant and insect species richness and diversity than hay meadows (95% confidence intervals are highlighted by shaded areas). The coloured dots denote the level of diversity assessed for the number of sampled units per meadow type (i.e. 30 for hay meadows, 16 for pastures).

At equal levels of sampling effort species richness, Shannon and Simpson diversity, were significantly lower in intensive pastures than in hay meadows for both plants and their pollinators (95% confidence intervals for both plant and pollinator diversity did not overlap between management types; Fig 1). Even when sampling effort was doubled the richness and diversity indices remained unchanged, plateauing near the interpolated value.

**Fig 2:**
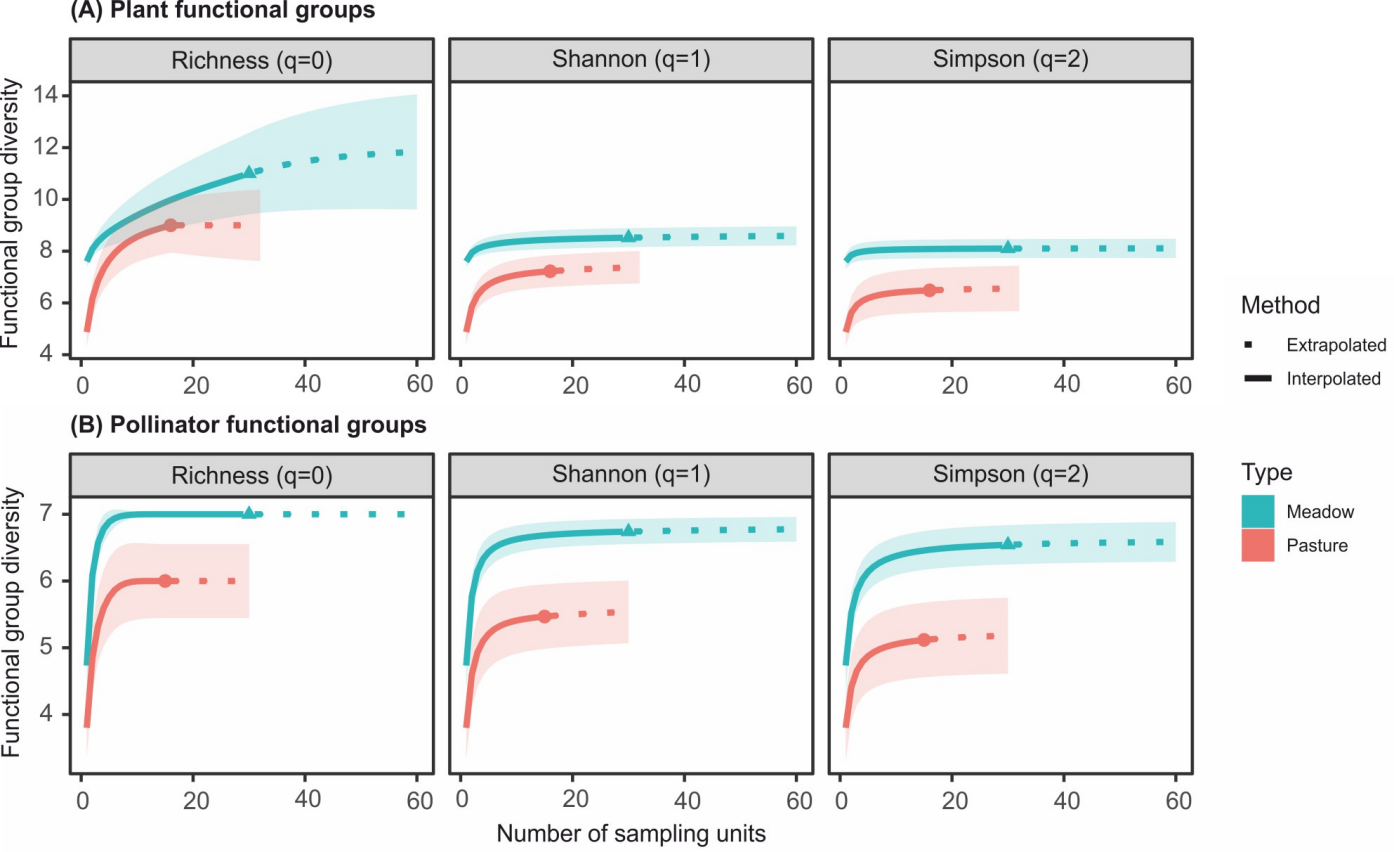
Differences in functional group richness and diversity between intensive pastures and traditional hay meadows. Sampling-unit (i.e. transects) based interpolation (continuous lines) and extrapolation (dashed lines, up to double the number of sampling units) of (A) plant and (B) pollinator functional group diversity using Hill numbers. Plant functional group richness was similar between hay meadows and pastures, while insect functional richness and plant and insect functional group diversity were significantly lower in intensive pastures (95% confidence intervals are highlighted by shaded areas). The coloured dots denote the level of functional group diversity assessed for the number of sampled units per meadow type (i.e. 30 for hay meadows, 16 for pastures).

Richness of plant functional group was not significantly different between management types, whereas pollinators were found to be significantly less functionally rich in pastures than in hay meadows. Both plants and pollinators had significantly lower Shannon and Simpson functional diversity in intensive pastures than in hay meadows (Fig 2). Extrapolating to double the sampling effort did not alter these patterns.

### Patterns of taxonomic and functional dissimilarity and dominance

Intensive pastures and hay meadows shared 19 of the 67 plant species (28.36%), while 11 species were unique to pastures (16.42%) and 37 to hay meadows (55.22%). The dissimilarity analysis thus showed clear differences among intensive pastures and hay meadows. These differences were mainly determined by turnover of species among management types rather than nestedness (ß_SOR_ = 0.5; ß_SIM_ = 0.456; ß_SNE_ = 0.04).

The comparison of the dominance patterns of plants revealed significant differences between intensive pastures and hay meadows (non-parametric ANOVA type test, F = 5.14, p = 0.04); pastures were thereby clearly dominated by a single species, *T*. *repens*. The abundance (percent relative cover) of *T*. *repens* was significantly lower in hay meadows (lower 5.9%) than in pastures (70.9%) (ssnonpartest, p<0.05) (see S1 Fig visualizing the 10 most dominant plant species for each management type).

Intensive pastures and hay meadows shared 30 of the 108 insect species (27.78%, 8 Hymenoptera, 17 Diptera, 5 Lepidoptera). Fourteen species were found only in intensive pastures (12.96%, 3 Hymenoptera, 10 Diptera, 1 Lepidoptera) and 63 were found only in hay meadows (58.33%, 8 Hymenoptera, 41 Diptera, 14 Lepidoptera). The dissimilarity analysis reflected these patterns, highlighting relatively high species dissimilarity between grassland management types, which appeared to be determined to a similar degree by species turnover and nestedness (ß_SOR_ = 0.58; ß_SIM_ = 0.34; ß_SNE_ = 0.24). Despite overall large shifts in the relative frequency of pollinators between intensive pastures and hay meadows (i.e. *Apis mellifera* shifted from 6.27% in hay meadows to 40.23% in pastures, *Autographa gamma* from 12.14% to 21.47% and *Bombus lapidarius* from 4.57% to 8.12%, S2 Fig), pastures and hay meadows shared 5 of the 10 most frequent pollinators. Management types did thus not significantly differ in the dominance patterns of the 10 most frequent pollinators (non-parametric ANOVA type test, F = 1.28, p = 0.35) (S2 Fig).

**Fig 3:**
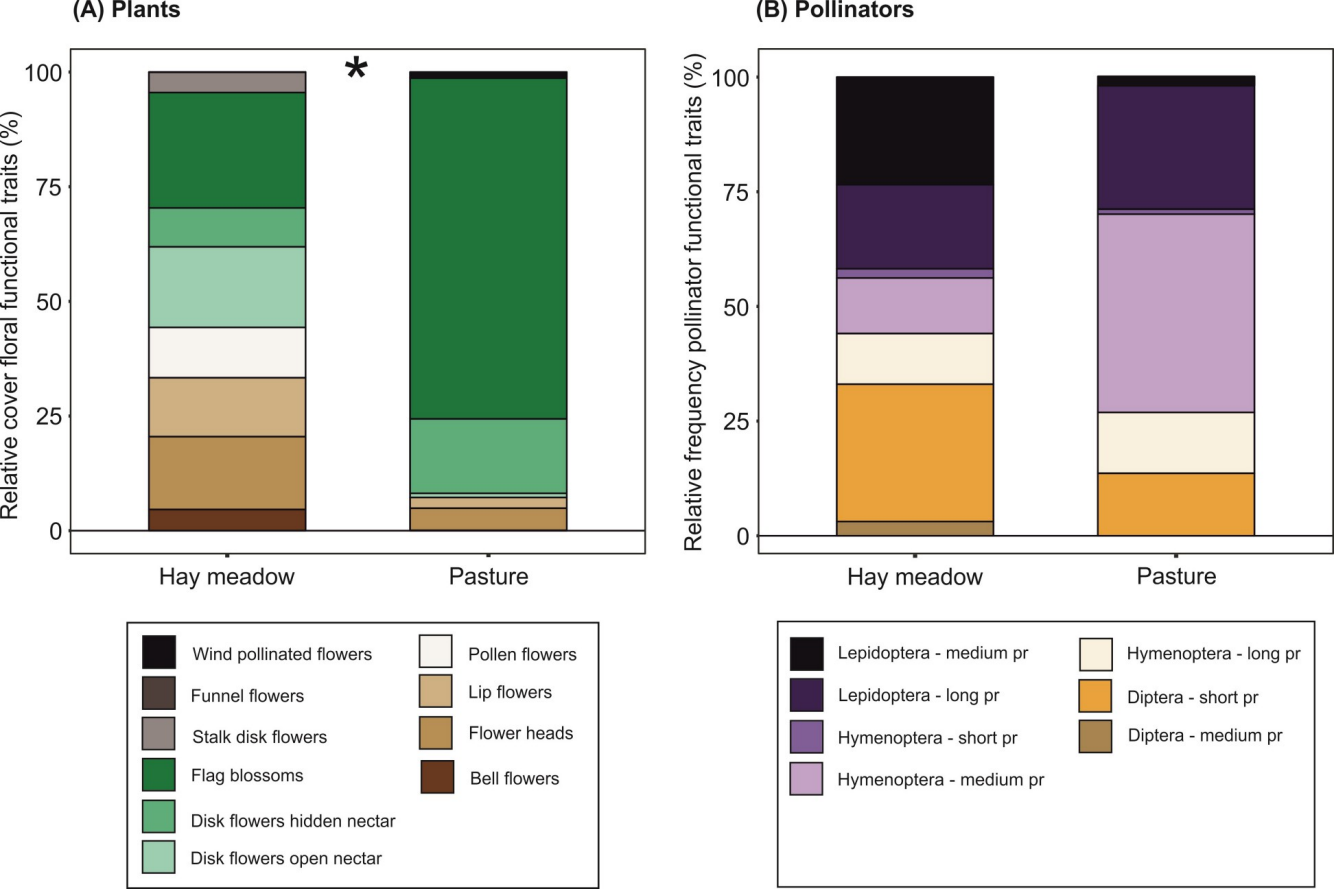
Differences in plant and pollinator functional group composition between intensive pastures and traditional hay meadows. Relative abundance of functional groups in hay meadows and pastures: (A) flowering plants (based on relative cover of floral functional traits); (B) pollinators (based on relative frequency of functional traits). (*) denotes significant differences between management types (non-parametric analysis of variance, p < 0.05).

The dissimilarity of functional groups among grassland management types was very low, both for plants and pollinators and determined solely by pastures having a nested subset of the functional groups found in hay meadows (plants: ß_SOR_ = 0.11; ß_SNE_ = 0.11; ß_SIM_ = 0; pollinators: ß_SOR_ = 0.08; ß_SNE_ = 0.08; ß_SIM_ = 0). Intensive pastures were significantly more dominated by one functional group of plants (flag blossoms) compared to hay meadows (non-parametric ANOVA type test, F = 6.25, p ≤ 0.001) (Fig 3A). The dominance pattern of pollinator functional groups did not differ between management types (Fig 3B, non-parametric ANOVA type test, F = 3.19, p = 0.129).

### Plant-pollinator networks

**Fig 4:**
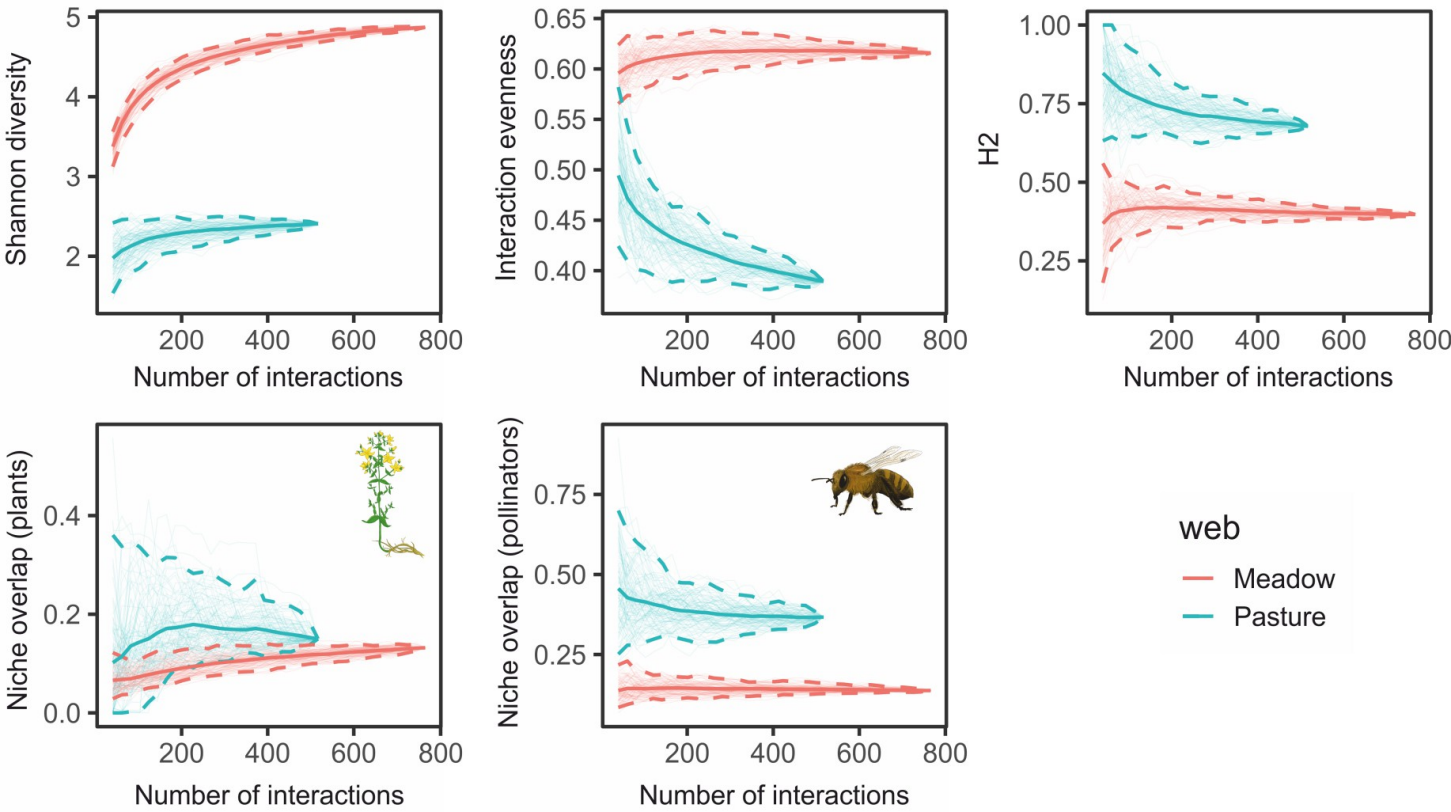
Plant-pollinator network structure. Interaction-based rarefaction curves comparing network metrics between hay meadows (blue) and pastures (pink). Shaded areas represent 95% confidence intervals. Plant and pollinator icons mark metrics calculated for either one of the two trophic levels, while graphs without icons were calculated for both trophic levels (drawings by S. C. Herbst and L. P. Sittel, CC BY-SA 4.0).

The diversity and evenness of interactions was significantly different (non-overlapping 95% confidence intervals) between intensive pastures and hay meadows, pastures having lower interaction diversity and lower interaction evenness of plant and pollinator species (Figs 4 and S3). Pasture networks were significantly more specialized than hay meadows. Plants occurring in intensive pastures were similar in the breath of their pollinator niche compared to hay meadow (results not shown), but pollinators showed a significantly higher niche overlap in pastures compared to hay meadows (Fig 4).

The modularity analysis revealed a decrease in the number of compartments in pastures, in comparison to hay meadows. The pasture network could thus be compartmentalized into only four modules with a likelihood (M) of 0.23. This stands in contrast to the hay meadow network which could be subdivided into six modules with a likelihood (M) of 0.45. Two of the modules within pastures contained just one plant and one pollinator species, while the other two contained 4.5±2.5 plant and 21±1 pollinator species. Modules in the hay meadow network comprised a mean (±SD) of 4.83±1.6 plant and 15.5±6.4 pollinator species. When the functional traits of both plants and pollinator were superimposed upon the modular structure of the pastures and hay meadows, the clustering of functional traits of plants and their connected pollinators could be visualized (S4 Fig). For instance, in pastures, one module contained a single plant species, *T*. *repens*, with relatively deep floral tubes associated with long to medium tongued Hymenoptera and Lepidoptera (S4 Fig). This species was part of a functionally similar module in hay meadows, albeit it had more interaction partners in pastures (S3 and S4 Figs).

#### The role of dominant species within networks

**Fig 5:**
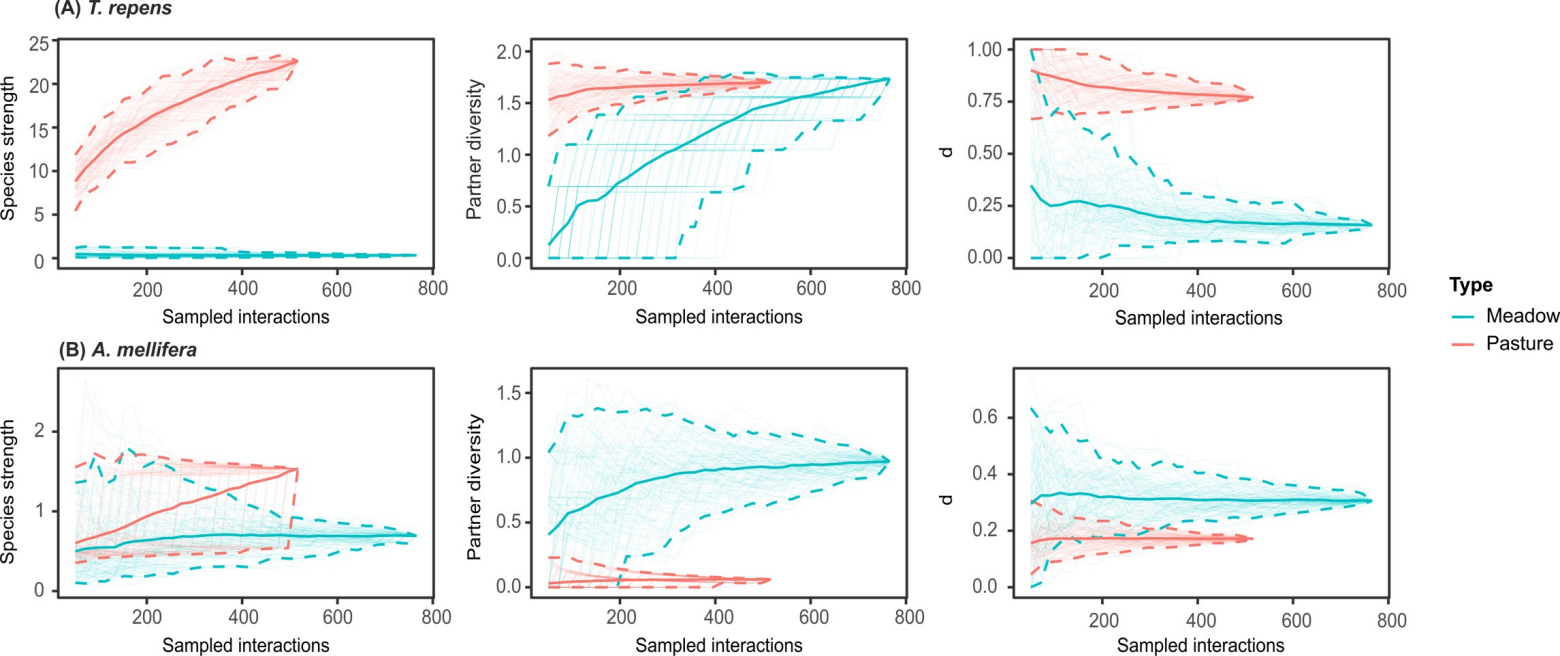
The role of species within plant-pollinator networks. Species level metrics for (A) *T*. *repens* and (B) *A*. *mellifera*. Comparison of species-level network metrics between hay meadows (blue) and pastures (pink) rarefied for pooled interactions. Shaded areas represent 95% confidence intervals.

The differentiation of the role individual species play in intensive pastures and hay meadows becomes apparent when species strength, partner diversity and d’ are analysed. *Trifolium repens*, the most dominant species in the pastures, shows significantly higher species strength and specialization in intensive pastures than in hay meadows, but similar values for partner diversity (Fig 5). In comparison to all other co-occurring plant species in pastures, *T*. *repens* became the species with the highest value of species strength and had mostly higher values of partner diversity and species level specialization. In hay meadows, *T*. *repens* had one of the lowest values of species strength (S5 Fig). The insect species which most frequently visited *T*. *repens*, *A*. *mellifera*, also largely changed its role in the network across grassland management types, with a non-significant trend for higher species strength in pastures and significantly lower values of partner diversity and specialization values in pastures compared to hay meadows (S6 Fig). Other frequent pollinators, such as *A*. *gamma* and species of the genus *Bombus* also had higher values of species strength in intensive pastures compared to hay meadows, and some of the lowest values for partner diversity and species level specialization in comparison to all pollinator species within pastures (S6 Fig).

## Discussion

It is known that intensive grazing can act as a strong filter, reducing plant diversity and altering community composition, but the parallel effect of grazing on functional groups, insects and plant-insect interactions has received less attention (but see [27, 38, 71, 72]). Our results show that, in contrast to traditional hay meadows, intensive pasture management reduces the taxonomic and functional diversity of pollinating insects and strongly shifts community composition towards the dominance of a single plant species with specialized floral traits. As the majority of the interactions in the intensive pastures shifted towards this single specialized plant species, its importance within the network increased and plant-pollinator networks became more specialized and less modular, features that have been linked with lower robustness of networks towards future perturbations [73–75]. Our results thus contribute to understanding the impact of intensive grazing on Europe’s species rich, semi-natural grasslands.

The observed loss of diversity of both plant and pollinators in intensive pastures is likely due to the high intensity of the pasture management [37, 38], as low-intensity grazing is known to produce similarly diverse plant communities as traditionally managed hay meadows [12]. We show strong shifts in community composition of plants, which were mostly attributed to species turnover and changes in evenness. Plants typically associated with hay meadows were replaced by grazing, trampling and nutrient tolerant species in pastures, and pasture sites were clearly dominated by a single species, *T*. *repens*. Changes in pollinator community composition were due to both species turnover and nestedness. Typical taxa for traditional hay meadows, such as the butterfly genus *Thymelicus*, were lost from pastures, and these were somewhat replaced by various groups of flies. These could, however, not compensate either in numbers, or in their functional role the loss of hay meadow species. Pastures were dominated by species such as *Apis mellifera*, *Autographa gamma* and *Bombus lapidarius*, which were the main visitors of *T*. *repens* in both management types. Our study thus supports previous research which has shown that intensive grazing can reduce both plant and insect species diversity and shift community composition towards the dominance of only a few taxa (e.g. [30, 35, 76], but see [77]).

Far less was previously known about the impact of intensive grazing on functional traits related to pollination, such as flower type [78–81]. We found that as plant communities became highly dominated by a single species, their functional composition changed as well. Thus intensive pastures were dominated by flag-type flowers, such as those of *T*. *repens*. This species is highly adapted to intensive grazing, trampling and nutrient input. Its flowers are of medium size and their nectar is hidden within the floral tube, favouring the access of long- to medium tongued Hymenoptera [26, 82, 83]. Thus the shift towards a species highly adapted to intensive pastures was, in the present case, also associated with the shift towards more specialized floral traits. Such shifts in plant dominance have been previously reported [19, 30, 81], however, their effects on the pollinator trophic level were previously largely unexplored, partly because the response of floral functional traits to intensive management has rarely been taken into consideration ([20, 21], but see [38]).

Intensive pastures were found to have lower functional diversity of pollinators than hay meadows, mainly due to the loss of a single functional group, Diptera species with medium proboscis length. Considering the strong shift in plant functional groups, the comparatively reduced response in pollinator functional diversity in intensive pastures suggests that species loss occurred quite evenly across pollinator functional groups, possibly because plant functional groups other than the dominating flag-type flowers were more generalized. This is supported by the fact that the second most abundant plant functional group in intensive pastures was that defined by disk flowers with hidden nectar, flowers which are accessible to many short and medium tongued pollinators. Alternatively, the fact that some of the visitors of the flag-type flowers were short tongued flies, which are unlikely to act as pollinators, suggests that our assessment of pollinator functional diversity might overestimate the true diversity of functional groups actually involved in pollination [68]. Such a bias, resulting from observing visitation rather than actual pollination events, may however not be very strong, as we found functional matching between most interacting plants and pollinators. This was also the case for the most common visitors of *T*. *repens* in intensive pastures, *A*. *mellifera*, *B*. *lapidarius* and *A*. *gamma*, species with medium to long proboscis and behaviours, well adapted to reach the nectar and pollen of flowers with deeper corolla tubes and more specialized flower structures. The dominance of *A*. *gamma*, *A*. *mellifera* and *B*. *lapidarius*, may thus be linked to the prevalence of *T*. *repens*, whose medium-deep inflorescences can be accessed by few other insects. Furthermore these species may be resistant to intensive grazing as they are able to fly long distances, forage on multiple host plant species, and build nests in man-made structures [84]. As no *A*. *mellifera* hives were observed in the close vicinity of our sampling sites, it is likely that their increased presence in the intensive pastures is due to the ample availability of a floral resource they can easily exploit.

In accordance to reductions in species diversity and shifts towards a single dominant taxa, *T*. *repens*, interaction networks in intensively managed pastures changed their topology [42, 85]. The only other plant-pollinator network study that considers the effects of intensive pasture management on flower functional traits was conducted in South-America, and found a shift towards the dominance of highly generalized Asteraceae plant species and minimal effects on pollinator communities and plant-pollinator networks [36]. In contrast, we found that a shift towards a more specialized plant species led to our pasture networks having low diversity of interactions, which were unevenly distributed towards a few partners. Thus species within intensive pastures relied on only a few interaction partners, which they increasingly shared with other species, as indicated by the high levels of network specialization and niche overlap [60]. These patterns likely reflect the decrease in species diversity in pastures and the wiring of interactions predominantly towards the single abundant resource available, the flowers of *T*. *repens* [39, 86]. The rewiring of interaction towards *T*. *repens* also led to a reduction of modularity from several relatively large modules in hay meadows, to just two main modules in pastures. Such simplified networks with lower diversity of species and interactions, higher specialization and niche overlap and lower modularity are expected to be less robust to perturbations [27, 71, 86, 87]. This is underlined by the fact that the loss or decline of a single species, of *T*. *repens* could lead to the collapse of the network, unless other flowering species take over its role. In contrast, in the taxonomically and functionally diverse hay meadows the most abundant plant and pollinator species were relatively evenly distributed among modules, a pattern which is likely to further contribute to the stability of hay meadow networks.

Species level analyses confirmed the increasing importance of *T*. *repens* and its pollinators in intensive pastures. Thus, the species strength and specialization of *T*. *repens*, increased in pastures, while partner diversity remained the same. The lack of effect on partner diversity is because *T*. *repens*, when correcting for network size, interacts with a similar proportion of pollinators. Furthermore, the species maintained mostly the same interactions among the two grassland management types. In pastures, *T*. *repens* became more specialized, not because it interacted with fewer species, but because the species it interacted with became almost exclusively dependent on it. As a result, *T*. *repens* is more important to network structure (i.e., species strength) in pastures.

The implications of this study, together with that of [36], are that the effects of grassland management intensification may depend on the functional traits of the dominant plant species. In Europe, intensive pastures are often characterized by higher abundance of Fabaceae (i.e. [30]), and the effect of the dominance of this plant group on higher trophic levels may therefore be broadly applicable across Europe. However, in order to better understand the generality of our results, more studies are needed that cover broader temporal and spatial sampling grains. For example, it is possible that intense grazing might have different effects at different temporal grains, as dominance patterns could change through time (e.g., [88]). A better understanding of how intensive grazing influences the structure of plant-pollinator networks can be achieved with more studies like this one across Europe, in grasslands with different baseline environmental characteristics (productivity, water availability, vegetation) and with other management features (e.g., type of grazing animals). The effects of intensive grazing on plant-pollinator networks will depend on its effects on the taxonomic and functional diversity and composition of both plants and pollinators, and this may vary predictably with grazing intensity. Finally our understanding would be improved by having information about the efficiency of the recorded visitors in transferring pollen (i.e. [68]). Assessments of pollen transfer patterns could provide essential information about the role of particular species in determining the coherence of networks, information which could support grassland conservation.

## Conclusions

Our results suggest that the response of plant-pollinator networks to intensive grazing could depend on the functional traits of the few dominant species adapted to permanent pastures. Our study joins a limited body of research on the effect of intensive grazing on plant-pollinator networks (i.e. [27, 36, 38, 71, 89]), and provides insights into some of the potential mechanisms that drive changes of ecological communities under intensive grassland management. Grazing is an essential management tool in conservation in Europe (i.e. [90, 91]). While moderate grazing plays an important role in maintaining the diversity of species and their interactions (i.e. [27]), intensive grazing is one of the major threats to highly diverse hay meadows in Central and Eastern Europe [16, 92, 93]. The extremely high stocking rates, continuous grazing throughout the year and lack of consideration for the boundaries of protected areas is likely having catastrophic consequences for the structure and stability of diverse plant-pollinator networks. However, so far, most evidence on the effect of intensive grazing in these regions has been anecdotal or limited to a few groups of species [18, 94]. If the diverse landscape of Central and Eastern Europe is to be conserved this knowledge gap needs to be urgently addressed. Our study, together with that of [36], thereby indicates that the threat of intensive grazing to plants, pollinators and their interactions could be potentially predicted and even ameliorated by considering the functional composition of the community. This study highlights that a synthetic understanding of how anthropogenic land use influences higher trophic levels and interaction networks requires explicit consideration of plant functional traits related to pollination.

## Supporting information

S1 Table Overview sampling sites. Details of the management type (italics denote the names used in all figure legends), locality, coordinates and altitude of the five studied grasslands.
(DOCX)

S2 Table Overview plant taxa. Details of all flowering plants from the five grasslands included in this study, their taxonomic classification, floral functional traits and ecological tolerance values for grazing, trampling and nitrogen. Tolerance values are coded as follows: intolerant (1); intolerant to sensitive (2); sensitive (3); sensitive to moderately tolerant (4); moderately tolerant (5); moderately tolerant to well tolerant (6); well tolerant (7); well tolerant to very tolerant (8); very tolerant (9). Management type indicates weather plant species were found exclusively in extensive hay meadows (M), intensive pastures (P) or both (M+P). NA denotes species with missing data.
(DOCX)

S3 Table Overview pollinator taxa. Details of all pollinators collected from the five grasslands included in this study, their taxonomical classification and functional traits (proboscis lengths were summarized into 3 groups: short, medium and long; NA denotes missing data). For Diptera information on the length of the proboscis was only available for Syrphidae. Management type indicates whether species were found exclusively in extensive hay meadows (M), intensive pastures (P) or both (M+P).
(DOCX)

S1 Fig Examples of main floral functional groups. **(A)** *Galium mollugo*—Disk flowers with open nectar (https://www.botanik-seite.de, René Rausch, CC BY 4.0). (B) *Leucanthemum vulgare*–flower heads (Demetra Rakosy, CC BY 4.0). (C) *Cerastium holosteoides*–Disk flowers with hidden nectar (https://www.naturadb.de/pflanzen/stellaria-holostea/, Der Michelis, CC 0.0). (D) *Prunella vulgaris*–Lip flowers (www.wikipedia.com, Georg Buzin, CC BY 4.0). (E) *Plantago media*–Pollen flowers (Demetra Rakosy, CC BY 4.0). (F) Campanula patula–Bell flowers (Demetra Rakosy, CC BY 4.0). (G) *Trifolium repens*–Flag blossom (www.wikipedia.com, Vinayaraj, CC BY 4.0). (H) *Dianthus deltoides–*Stalk disk flowers (www.wikipedia.com, Robert Flogaus-Faust, CC BY 4.0). (I) *Convolvulus arvensis*–Funnel flowers (https://commons.wikimedia.org/, Michel_Langeveld, CC BY 4.0).
(TIF)

S2 Fig Differences in plant and pollinator species composition between grassland management types. Visualization of the 10 most abundant flowering plant species (based on relative cover) and 10 most frequent pollinator species (based on relative frequency) in (A) hay meadows and (B) pastures. Plant species are colour coded in shades of green, pollinator species in orange. (*) denotes species shared between hay meadows and pastures. The non-parametric analysis of variance revealed significant differences between the composition and abundance of the most common plant species in meadows and pastures (p < 0.05). For pollinators no significant differences between management types were found. Plant and insect icons drawn by S. C. Herbst and L. P. Sittel (CC BY-SA 4.0).
(TIF)

S3 Fig Plant-pollinator interaction networks. Networks for (A) hay meadows and (B) pastures. Pollinator species are shown by genus for visualization purposes only; all statistical analyses were performed at the species level. Red field margins highlight insects species which interact with *T*. *repens*, while red filled fields highlight *A*. *mellifera*, *A*. *gamma* and several species of *Bombus* which became the main visitors of *T*. *repens* in intensive pastures. Plants are demarked by green filled fields.
(TIF)

S4 Fig Plant-pollinator network modularity. Module structure of (A) hay meadows and (B) pastures illustrating the taxonomic and functional composition of each module. Grey shaded squares indicate weighted interactions (darker colours indicate higher interaction frequency). Colour codes indicate the functional group for each species and these colours follow those in Fig 3 (grey denotes species whose functional traits could not be assessed, most of which were Diptera). Bold names indicate the 10 most abundant plant and pollinator species.
(ZIP)

S5 Fig Species level metrics of T. repens in comparison to all other plants in the networks of (A) hay meadows and (B) pastures. Metrics have been rarefied for pooled interactions. Shaded areas represent 95% confidence intervals.
(TIF)

S6 Fig Species level metrics of A. mellifera, A. gamma and B. lapidarius in comparison to all other pollinator species in the networks of: (A) hay meadows and (B) pastures. Metrics have been rarefied for pooled interactions. Shaded areas represent 95% confidence intervals. Besides A. mellifera, A. gamma and B. lapidarius which were the most frequent pollinators of T. repens, we highlighted also other Bombus species frequently observed on the flowers for reference.
(TIF)

S1 Appendix Indirect measure of land-use intensity. (DOCX)
