# Supplementary material for: Intensive grazing alters the diversity, composition and structure of plant-pollinator interaction networks in Central European grasslands
Source: PLoS One. 2022 Mar 11;17(3):e0263576. doi: 10.1371/journal.pone.0263576 (PMC8916670; doi:10.1371/journal.pone.0263576)
Supplement: S1 Table — Details of the management type (italics denote the names used in all figure legends), locality, coordinates and altitude of the five studied grasslands. (DOCX) [file pone.0263576.s001.docx]

**S1 Table.** **Overview sampling sites.** Details of the management type (italics denote the names used in all figure legends), locality, coordinates and altitude of the five studied grasslands.

| **Management type** | **Locality** | **Coordinates** | **Altitude** |
| --- | --- | --- | --- |
| Extensive hay *meadow 1* | Hermanovice | 50.201429, 17.412062 | 750m |
| Extensive hay *meadow 2* | Horni Grunt | 50.210563, 17.349508 | 730m |
| Extensive hay *meadow 3* | Mnichov | 50.150308, 17.379851 | 608m |
| Intensive *pasture 1* | Jarnoltowek | 50.275333, 17.406683 | 390m |
| Intensive *pasture 2* | Hermanovice | 50.199440, 17.393010 | 695m |
