# Supplementary material for: Intensive grazing alters the diversity, composition and structure of plant-pollinator interaction networks in Central European grasslands
Source: PLoS One. 2022 Mar 11;17(3):e0263576. doi: 10.1371/journal.pone.0263576 (PMC8916670; doi:10.1371/journal.pone.0263576)
Supplement: S2 Table — Details of all flowering plants from the five grasslands included in this study, their taxonomic classification, floral functional traits and ecological tolerance values for grazing, trampling and nitrogen. Tolerance values are coded as follows: intolerant (1); intolerant to sensitive (2); sensitive (3); sensitive to moderately tolerant (4); moderately tolerant (5); moderately tolerant to well tolerant (6); well tolerant (7); well tolerant to very tolerant (8); very tolerant (9). Management type indicates weather plant species were found exclusively in extensive hay meadows (M), intensive pastures (P) or both (M+P). NA denotes species with missing data. (DOCX) [file pone.0263576.s002.docx]

**S2 Table. Overview plant taxa.** Details of all flowering plants from the five grasslands included in this study, their taxonomic classification, floral functional traits and ecological tolerance values for grazing, trampling and nitrogen. Tolerance values are coded as follows: intolerant (1); intolerant to sensitive (2); sensitive (3); sensitive to moderately tolerant (4); moderately tolerant (5); moderately tolerant to well tolerant (6); well tolerant (7); well tolerant to very tolerant (8); very tolerant (9). Management type indicates weather plant species were found exclusively in extensive hay meadows (M), intensive pastures (P) or both (M+P). NA denotes species with missing data.

| **Management type** | **Family** | **Plant** | **Simplified functional traits (after Kügler)** | **Grazing tolerance** | **Trampling tolerance** | **Nitrogen tolerance** |
| --- | --- | --- | --- | --- | --- | --- |
| M+P | Apiaceae | *Aegopodium podagraria* | Disk flowers  with open nectar | 2 | 4 | 8 |
| M | Apiaceae | *Angelica*  *sylvestris* | Disk flowers  with open nectar | 2 | 2 | 4 |
| M | Apiaceae | *Anthriscus*  *sylvestris* | Disk flowers  with open nectar | 3 | 3 | 8 |
| M+P | Apiaceae | *Chaerophyllum aromaticum* | Disk flowers  with open nectar | NA | NA | NA |
| M | Apiaceae | *Pimpinella*  *saxifraga* | Disk flowers  with open nectar | 5 | 5 | 2 |
| P | Boraginaceae | *Symphytum officinale* | Bell flowers | 4 | 4 | 8 |
| M | Campanulaceae | *Campanula*  *patula* | Bell flowers | 2 | 2 | 5 |
| M | Campanulaceae | *Campanula persicifolia* | Bell flowers | 4 | 2 | 3 |
| M | Campanulaceae | *Campanula rapunculoides* | Bell flowers | 4 | 4 | 4 |
| M+P | Caryophyllaceae | *Cerastium holosteoides* | Disk flowers  with hidden nectar | 4 | 4 | 5 |
| M | Caryophyllaceae | *Dianthus*  *deltoides* | Stalk disk flowers | 4 | 4 | 2 |
| M | Caryophyllaceae | *Lychnis*  *flos-cuculi* | Stalk disk flowers | 2 | 2 | 0 |
| M | Caryophyllaceae | *Silene*  *vulgaris* | Stalk disk flowers | 2 | 2 | 4 |
| M+P | Caryophyllaceae | *Stellaria*  *graminea* | Disk flowers  with hidden nectar | 5 | 5 | 3 |
| M | Clusiaceae | *Hypericum maculatum* | Pollen flowers | 3 | 3 | 2 |
| M+P | Compositae | *Achillea*  *millefolium* | Flower heads | NA | NA | NA |
| M+P | Compositae | *Alchemilla*  *spp.* | Disk flowers  with open nectar | NA | NA | NA |
| P | Compositae | *Bellis*  *perennis* | Flower heads | 8 | 8 | 6 |
| M | Compositae | *Centaurea pseudophrygia* | Flower heads | 2 | 3 | 4 |
| M+P | Compositae | *Crepis*  *biennis* | Flower heads | 2 | 2 | 5 |
| P | Compositae | *Crepis*  *capillaris* | Flower heads | 5 | 5 | 4 |
| M | Compositae | *Crepis*  *mollis* | Flower heads | 3 | 3 | 5 |
| P | Compositae | *Erigeron*  *acris* | Flower heads | 2 | 2 | 2 |
| M | Compositae | *Hieracium*  *spp.* | Flower heads | NA | NA | NA |
| M+P | Compositae | *Hypochaeris radicata* | Flower heads | 3 | 3 | 3 |
| M | Compositae | *Leontodon*  *hispidus* | Flower heads | 5 | 5 | 6 |
| P | Compositae | *Leontodon autumnalis* | Flower heads | 7 | 7 | 5 |
| M | Compositae | *Leucanthemum vulgare* | Flower heads | 3 | 4 | 3 |
| M | Compositae | *Senecio*  *jacobaea* | Flower heads | 9 | 4 | 5 |
| P | Compositae | *Taraxacum officinalis* | Flower heads | 7 | 7 | 8 |
| M+P | Convolvulaceae | *Convolvulus*  *arvensis* | Funnel flowers | 4 | 4 | 0 |
| P | Cruciferae | *Capsella*  *bursa-pastoris* | Disk flowers  with hidden nectar | 7 | 6 | 6 |
| M | Dipsacaceae | *Knautia*  *arvensis* | Flower heads | 3 | 2 | 4 |
| M+P | Fabaceae | *Lathyrus*  *pratensis* | Flag blossoms | 2 | 3 | 6 |
| M | Fabaceae | *Lotus*  *corniculatus* | Flag blossoms | 4 | 4 | 3 |
| M | Fabaceae | *Trifolium*  *dubium* | Flag blossoms | 4 | 4 | 4 |
| M | Fabaceae | *Trifolium*  *medium* | Flag blossoms | 5 | 4 | 3 |
| M+P | Fabaceae | *Trifolium*  *pratense* | Flag blossoms | 4 | 4 | 0 |
| M+P | Fabaceae | *Trifolium*  *repens* | Flag blossoms | 8 | 8 | 6 |
| M | Fabaceae | *Vicia*  *cracca* | Flag blossoms | 1 | 2 | 0 |
| M+P | Fabaceae | *Vicia*  *sepium* | Flag blossoms | 1 | 2 | 5 |
| P | Geraniaceae | *Geranium*  *pratense* | Disk flowers  with hidden nectar | 2 | 2 | 7 |
| M | Lamiaceae | *Clinopodium*  *vulgare* | Lip flowers | 3 | 3 | 3 |
| M | Lamiaceae | *Pimpinella*  *major* | Lip flowers | 3 | 2 | 5 |
| M+P | Lamiaceae | *Prunella*  *vulgaris* | Lip flowers | 8 | 8 | 0 |
| M | Lamiaceae | *Thymus*  *pulegioides* | Lip flowers | 4 | 4 | 1 |
| M | Liliaceae | *Gladiolus*  *imbricatus* | Lip flowers | NA | NA | NA |
| P | Onagraceae | *Epilobium*  spp. | Disk flowers  with hidden nectar | 2 | 2 | 8 |
| M | Orchidaceae | *Gymnadenia conopsea* | Lip flowers | 3 | 2 | 3 |
| M | Orchidaceae | *Platanthera*  *bifolia* | Lip flowers | 4 | 2 | 0 |
| M | Orobanchaceae | *Euphrasia rostkoviana* | Lip flowers | 6 | 5 | 4 |
| M | Orobanchaceae | *Rhinanthus*  *minor* | Lip flowers | 8 | 3 | 3 |
| M+P | Plantaginaceae | *Plantago*  *lanceolata* | Wind pollinated flowers | 6 | 6 | 0 |
| P | Plantaginaceae | *Veronica beccabunga* | Lip flowers | 2 | 2 | 6 |
| M+P | Plantaginaceae | *Veronica chamaedrys* | Lip flowers | 6 | 6 | 6 |
| M | Plantaginaceae | *Veronica*  *officinalis* | Lip flowers | 4 | 4 | 4 |
| M | Polygalaceae | *Polygala*  *vulgaris* | Flag blossoms | 4 | 4 | 2 |
| P | Polygonaceae | *Rumex*  *obtusifolius* | Wind pollinated flowers | 7 | 3 | 9 |
| M | Primulaceae | *Lysimachia*  *vulgaris* | Pollen flowers | 2 | 2 | 0 |
| M+P | Ranunculaceae | *Ranunculus*  *acris* | Disk flowers  with hidden nectar | 5 | 6 | 0 |
| M+P | Ranunculaceae | *Ranunculus*  *repens* | Disk flowers  with hidden nectar | 7 | 7 | 7 |
| M+P | Rosaceae | *Potentilla*  *anserina* | Disk flowers  with hidden nectar | 9 | 9 | 7 |
| M | Rosaceae | *Potentilla*  *erecta* | Disk flowers  with hidden nectar | 4 | 5 | 2 |
| M | Rubiaceae | *Galium*  *mollugo* | Disk flowers  with open nectar | 3 | 3 | 0 |
| M | Rubiaceae | *Galium*  *pumilum* | Disk flowers  with open nectar | 4 | 4 | 2 |
| M | Rubiaceae | *Galium*  *saxatile* | Disk flowers with open nectar | 7 | 7 | 2 |
| M | Scrophulariaceae | *Verbascum*  spp. | Lip flowers | NA | NA | NA |
