# Supplementary material for: Intensive grazing alters the diversity, composition and structure of plant-pollinator interaction networks in Central European grasslands
Source: PLoS One. 2022 Mar 11;17(3):e0263576. doi: 10.1371/journal.pone.0263576 (PMC8916670; doi:10.1371/journal.pone.0263576)
Supplement: S3 Table — Details of all pollinators collected from the five grasslands included in this study, their taxonomical classification and functional traits (proboscis lengths were summarized into 3 groups: short, medium and long; NA denotes missing data). For Diptera information on the length of the proboscis was only available for Syrphidae. Management type indicates whether species were found exclusively in extensive hay meadows (M), intensive pastures (P) or both (M+P). (DOCX) [file pone.0263576.s003.docx]

**S3 Table. Overview pollinator taxa.** Details of all pollinators collected from the five grasslands included in this study, their taxonomical classification and functional traits (proboscis lengths were summarized into 3 groups: short, medium and long; NA denotes missing data). For Diptera information on the length of the proboscis was only available for Syrphidae. Management type indicates whether species were found exclusively in extensive hay meadows (M), intensive pastures (P) or both (M+P).

| **Meadow type** | **Order** | **Family** | ***Species*** | **Functional.Group** |
| --- | --- | --- | --- | --- |
| M+P | Diptera | Anthomyiidae | *Delia sp.1* | NA |
| P | Diptera | Anthomyiidae | *Delia sp.2* | NA |
| M | Diptera | Anthomyiidae | *Delia sp.3* | NA |
| M | Diptera | Anthomyiidae | *Lasiomma sp.1* | NA |
| M | Diptera | Anthomyiidae | *Lasiomma sp.2* | NA |
| M | Diptera | Anthomyiidae | *Lasiomma sp.3* | NA |
| M | Diptera | Anthomyiidae | *Lasiomma sp.4* | NA |
| P | Diptera | Anthomyiidae | *Leucophora sp.1* | NA |
| M+P | Diptera | Anthomyiidae | *Paregle sp.1* | NA |
| M | Diptera | Anthomyiidae | *Paregle sp.3* | NA |
| M | Diptera | Anthomyiidae | *Pegomya sp.1* | NA |
| M+P | Diptera | Anthomyiidae | *Pegoplata sp.1* | NA |
| M | Diptera | Anthomyiidae | *Pegoplata sp.2* | NA |
| M+P | Diptera | Anthomyiidae | *Pegoplata sp.3* | NA |
| M | Diptera | Calliphoridae | *Bellardia sp. 1* | NA |
| P | Diptera | Calliphoridae | *Bellardia sp. 2* | NA |
| M+P | Diptera | Calliphoridae | *Bellardia sp. 3* | NA |
| M | Diptera | Calliphoridae | *Bellardia sp. 4* | NA |
| M | Diptera | Calliphoridae | *Bellardia sp. 5* | NA |
| P | Diptera | Calliphoridae | *Melanomya cf. nana* | NA |
| M | Diptera | Calliphoridae | *Pollenia sp.1* | NA |
| P | Diptera | Conopidae | *Sicus sp.1* | NA |
| P | Diptera | Drosphilidae | *Drosophilidae sp. 1* | NA |
| M | Diptera | Empididae | *Empis sp. 1* | NA |
| P | Diptera | Empididae | *Empis sp. 2* | NA |
| P | Diptera | Fanniidae | *Fannia sp. 1* | NA |
| M | Diptera | Muscidae | *Eudasyphora sp.1* | NA |
| M | Diptera | Muscidae | *Helina* sp. 1 | NA |
| M | Diptera | Muscidae | *Musca* sp.1 | NA |
| M | Diptera | Muscidae | *Phaonia* sp.1 | NA |
| M | Diptera | Muscidae | *Thricops* sp. 2 | NA |
| M | Diptera | Muscidae | *Thricops* sp.1 | NA |
| M | Diptera | Opomyzidae | *Geomyza* sp.1 | NA |
| M | Diptera | Rhagionidae | *Rhagio cf. tringarius* | NA |
| P | Diptera | Sarcophagidae | *Macronychia* sp. 1 | NA |
| M+P | Diptera | Sarcophagidae | *Sarcophagini* sp. 2 | NA |
| M | Diptera | Sarcophagidae | *Sarcophagini* sp. 4 | NA |
| M | Diptera | Sarcophagidae | *Sarcophagini* sp. 5 | NA |
| M | Diptera | Stratiomyidae | *Sargus* sp.1 | NA |
| M+P | Diptera | Syrphidae | *Cheliosa vulpina* | Short Proboscis |
| M | Diptera | Syrphidae | *Chrysosyrphus nasutus* | Short Proboscis |
| M | Diptera | Syrphidae | *Chrysotoxum bicinctum* | Short Proboscis |
| M+P | Diptera | Syrphidae | *Episyrphus balteatus* | Short Proboscis |
| M | Diptera | Syrphidae | *Eristalis hirta* | Medium Proboscis |
| M | Diptera | Syrphidae | *Eristalis nemorum* | Medium Proboscis |
| M | Diptera | Syrphidae | *Eristalis rupium* | Medium Proboscis |
| M | Diptera | Syrphidae | *Eristalis tenax* | Medium Proboscis |
| M+P | Diptera | Syrphidae | *Eupeodes corollae* | Short Proboscis |
| M | Diptera | Syrphidae | *Eupeodes lundbecki* | Short Proboscis |
| M | Diptera | Syrphidae | *Eupeodes luniger* | Short Proboscis |
| M | Diptera | Syrphidae | *Helophilus hybridus* | Medium Proboscis |
| M | Diptera | Syrphidae | *Helophilus pendulus* | Medium Proboscis |
| M | Diptera | Syrphidae | *Helophilus trivittatus* | Medium Proboscis |
| M | Diptera | Syrphidae | *Melanostoma mellinum* | NA |
| M+P | Diptera | Syrphidae | *Platycheirus albimanus* | Short Proboscis |
| P | Diptera | Syrphidae | *Scaeva dignota* | Short Proboscis |
| M | Diptera | Syrphidae | *Scaeva pyrastri* | Short Proboscis |
| M | Diptera | Syrphidae | *Sphaerophoria batava* | Short Proboscis |
| M+P | Diptera | Syrphidae | *Sphaerophoria interrupta* | Short Proboscis |
| M+P | Diptera | Syrphidae | *Sphaerophoria ruppellii* | Short Proboscis |
| M+P | Diptera | Syrphidae | *Sphaerophoria scripta* | Short Proboscis |
| M+P | Diptera | Syrphidae | *Sphaerophoria taeniata* | Short Proboscis |
| M | Diptera | Syrphidae | *Syrphus ribesii* | Short Proboscis |
| M+P | Diptera | Syrphidae | *Xanthandrus comtus* | Short Proboscis |
| M+P | Diptera | Tachinidae | *Minthodes* sp.1 | NA |
| M | Diptera | Tachinidae | Phasia *sp.1* | NA |
| M | Diptera | Tachinidae | *Voria cf. ruralis* | NA |
| M+P | Diptera | Tachinidae | *Zophomya cf. temula* | NA |
| P | Hymenoptera | Andrenidae | *Andrena flavipes* | Short Proboscis |
| M | Hymenoptera | Andrenidae | *Andrena hattorfiana* | Short Proboscis |
| M | Hymenoptera | Andrenidae | *Andrena niveata* | Short Proboscis |
| P | Hymenoptera | Andrenidae | *Andrena similis* | Short Proboscis |
| M+P | Hymenoptera | Apidae | *Apis mellifera* | Medium Proboscis |
| M+P | Hymenoptera | Apidae | *Bombus lapidarius* | Long Proboscis |
| M+P | Hymenoptera | Apidae | *Bombus pascuorum* | Medium Proboscis |
| M+P | Hymenoptera | Apidae | *Bombus pratorum* | Medium Proboscis |
| M+P | Hymenoptera | Apidae | *Bombus ruderarius* | Long Proboscis |
| M | Hymenoptera | Apidae | *Bombus soroeensis* | Medium Proboscis |
| M+P | Hymenoptera | Apidae | *Bombus sylvarum* | Long Proboscis |
| M+P | Hymenoptera | Apidae | *Bombus terrestris* | Long Proboscis |
| M | Hymenoptera | Apidae | *Sphecodes* sp.1 | Short Proboscis |
| M | Hymenoptera | Apidae | *Trachusa byssinum* | Medium Proboscis |
| M | Hymenoptera | Halictidae | *Lasioglossum albipes* | Short Proboscis |
| M | Hymenoptera | Halictidae | *Lasioglossum calceatum* | Short Proboscis |
| M+P | Hymenoptera | Halictidae | *Lasioglossum lativentre* | Short Proboscis |
| P | Hymenoptera | Vespidae | *Polistinae* sp.1 | Short Proboscis |
| M | Hymenoptera | Vespidae | *Polistes nimpha* | Short Proboscis |
| M | Lepidoptera | Hesperidae | *Ochlodes sylvanus* | Long Proboscis |
| M | Lepidoptera | Hesperidae | *Thymelicus* spp. | Long Proboscis |
| M | Lepidoptera | Lycaenidae | *Lycaena hyppothoe* | NA |
| M+P | Lepidoptera | Noctuidae | *Autographa gamma* | Long Proboscis |
| M | Lepidoptera | Noctuidae | *Cerapteryx graminis* | Medium Proboscis |
| P | Lepidoptera | Nymphalidae | *Aglais io* | Long Proboscis |
| M+P | Lepidoptera | Nymphalidae | *Aphantopus hyperantus* | Medium Proboscis |
| M | Lepidoptera | Nymphalidae | *Argynnis adippe* | Long Proboscis |
| M | Lepidoptera | Nymphalidae | *Argynnis aglaja* | Long Proboscis |
| M | Lepidoptera | Nymphalidae | *Coenonympha glycerion* | Medium Proboscis |
| M | Lepidoptera | Nymphalidae | *Maniola jurtina* | Long Proboscis |
| M | Lepidoptera | Nymphalidae | *Melanargia galatea* | Long Proboscis |
| M+P | Lepidoptera | Pieridae | *Pieris brassicae* | Long Proboscis |
| M+P | Lepidoptera | Pieridae | *Pieris napi* | Long Proboscis |
| M+P | Lepidoptera | Pieridae | *Pieris rapae* | Medium Proboscis |
| M | Lepidoptera | Pyralidae | *Pyralidae* spp. | NA |
| M | Lepidoptera | Zygaenidae | *Adscita* spp. | Medium Proboscis |
| M | Lepidoptera | Zygaenidae | *Zygaena filipendulae* | Medium Proboscis |
| M | Lepidoptera | Zygaenidae | *Zygaena loti* | Medium Proboscis |
| M | Lepidoptera | Zygaenidae | *Zygaena vicia* | Medium Proboscis |
