# Supplementary figures and images for: Intensive grazing alters the diversity, composition and structure of plant-pollinator interaction networks in Central European grasslands
Source: PLoS One. 2022 Mar 11;17(3):e0263576. doi: 10.1371/journal.pone.0263576 (PMC8916670; doi:10.1371/journal.pone.0263576)

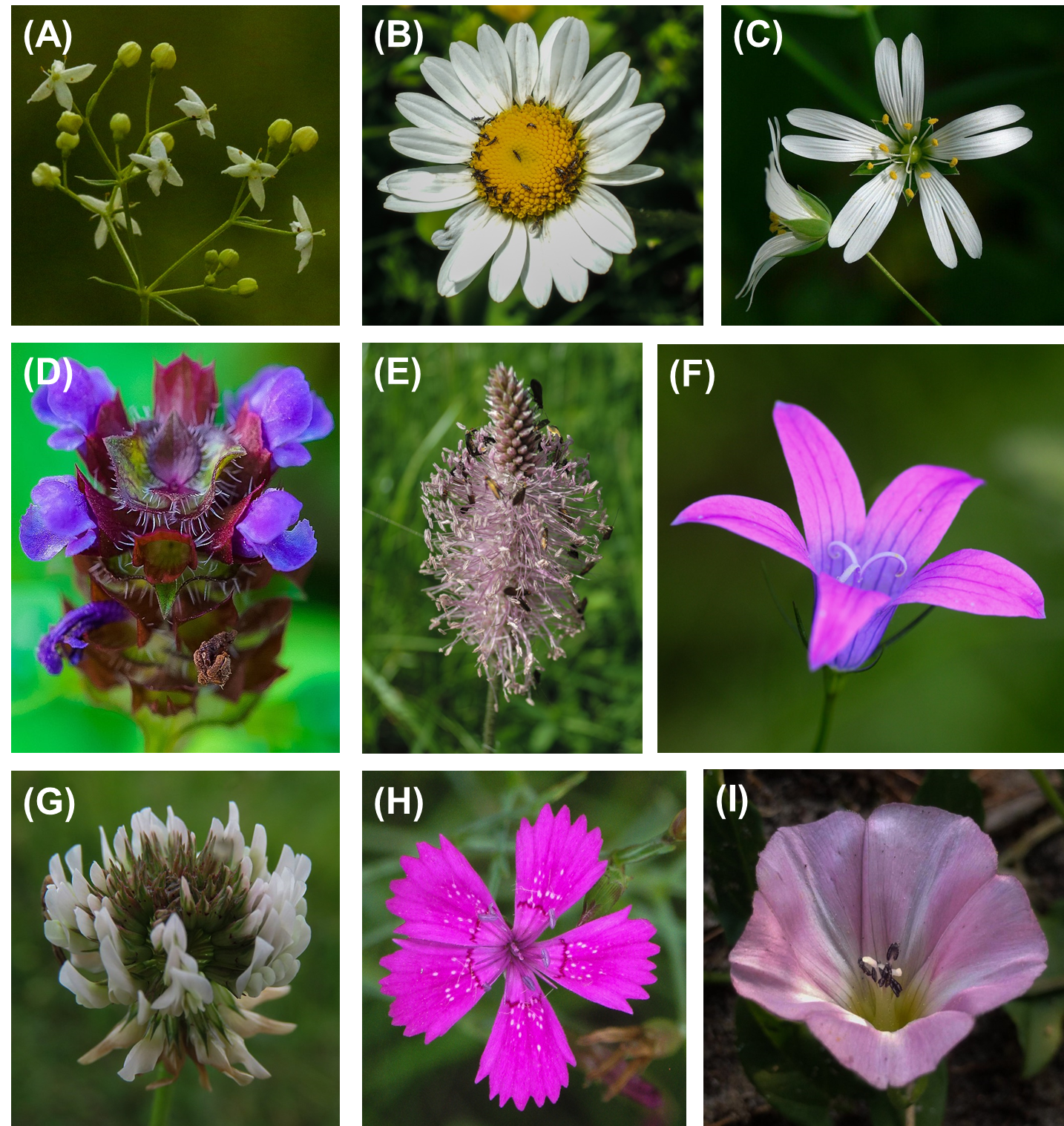

Supplement: S1 Fig — (A) Galium mollugo—Disk flowers with open nectar (https://www.botanik-seite.de, René Rausch, CC BY 4.0). (B) Leucanthemum vulgare–flower heads (Demetra Rakosy, CC BY 4.0). (C) Cerastium holosteoides–Disk flowers with hidden nectar (https://www.naturadb.de/pflanzen/stellaria-holostea/, Der Michelis, CC 0.0). (D) Prunella vulgaris–Lip flowers (www.wikipedia.com, Georg Buzin, CC BY 4.0). (E) Plantago media–Pollen flowers (Demetra Rakosy, CC BY 4.0). (F) Campanula patula–Bell flowers (Demetra Rakosy, CC BY 4.0). (G) Trifolium repens–Flag blossom (www.wikipedia.com, Vinayaraj, CC BY 4.0). (H) Dianthus deltoides–Stalk disk flowers (www.wikipedia.com, Robert Flogaus-Faust, CC BY 4.0). (I) Convolvulus arvensis–Funnel flowers (https://commons.wikimedia.org/, Michel_Langeveld, CC BY 4.0). (TIF) [file pone.0263576.s004.tif]

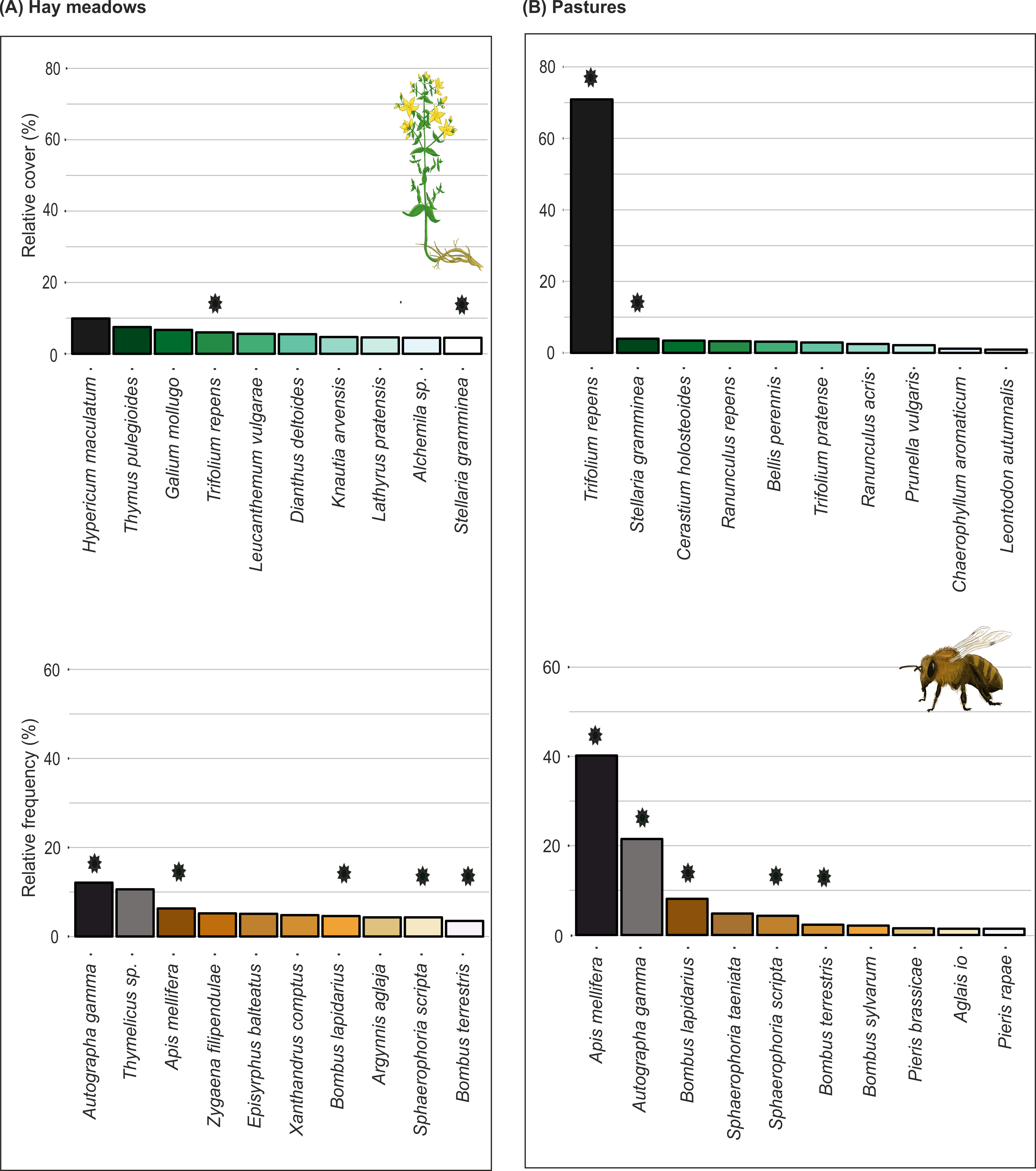

Supplement: S2 Fig — Visualization of the 10 most abundant flowering plant species (based on relative cover) and 10 most frequent pollinator species (based on relative frequency) in (A) hay meadows and (B) pastures. Plant species are colour coded in shades of green, pollinator species in orange. (*) denotes species shared between hay meadows and pastures. The non-parametric analysis of variance revealed significant differences between the composition and abundance of the most common plant species in meadows and pastures (p < 0.05). For pollinators no significant differences between management types were found. Plant and insect icons drawn by S. C. Herbst and L. P. Sittel (CC BY-SA 4.0). (TIF) [file pone.0263576.s005.tif]

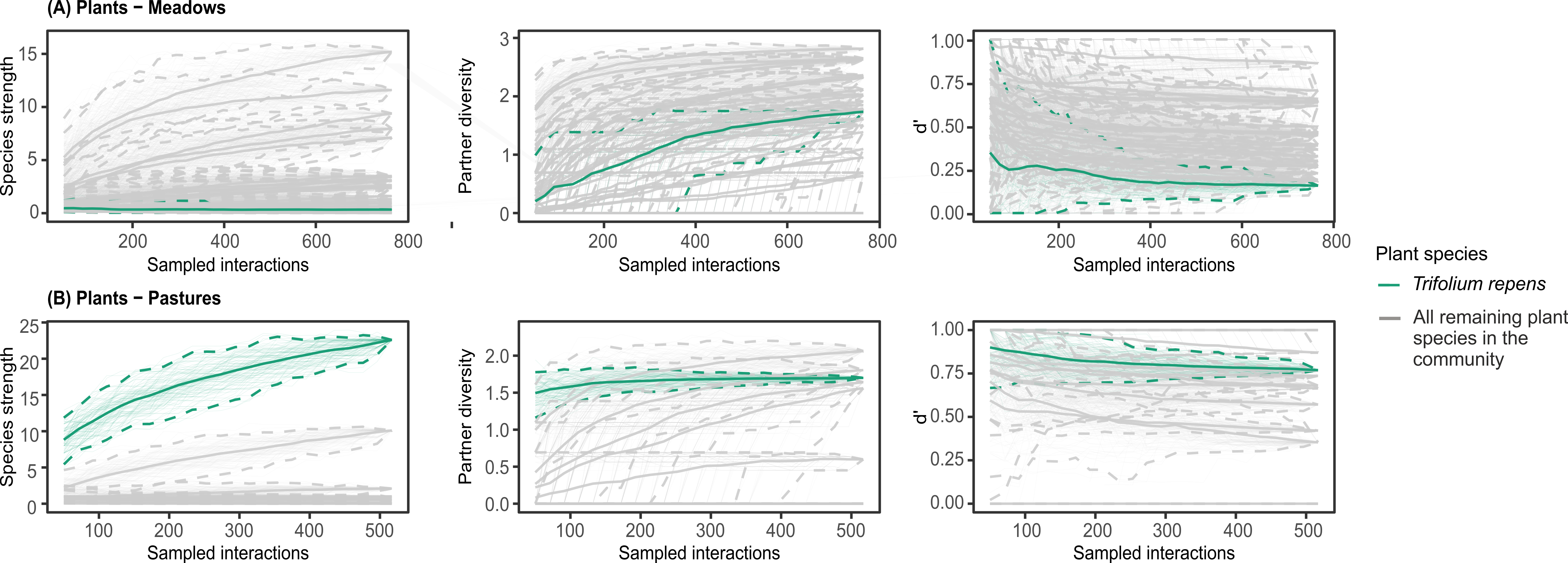

Supplement: S5 Fig — Species level metrics of T. repens in comparison to all other plants in the networks of (A) hay meadows and (B) pastures. Metrics have been rarefied for pooled interactions. Shaded areas represent 95% confidence intervals. (TIF) [file pone.0263576.s008.tif]

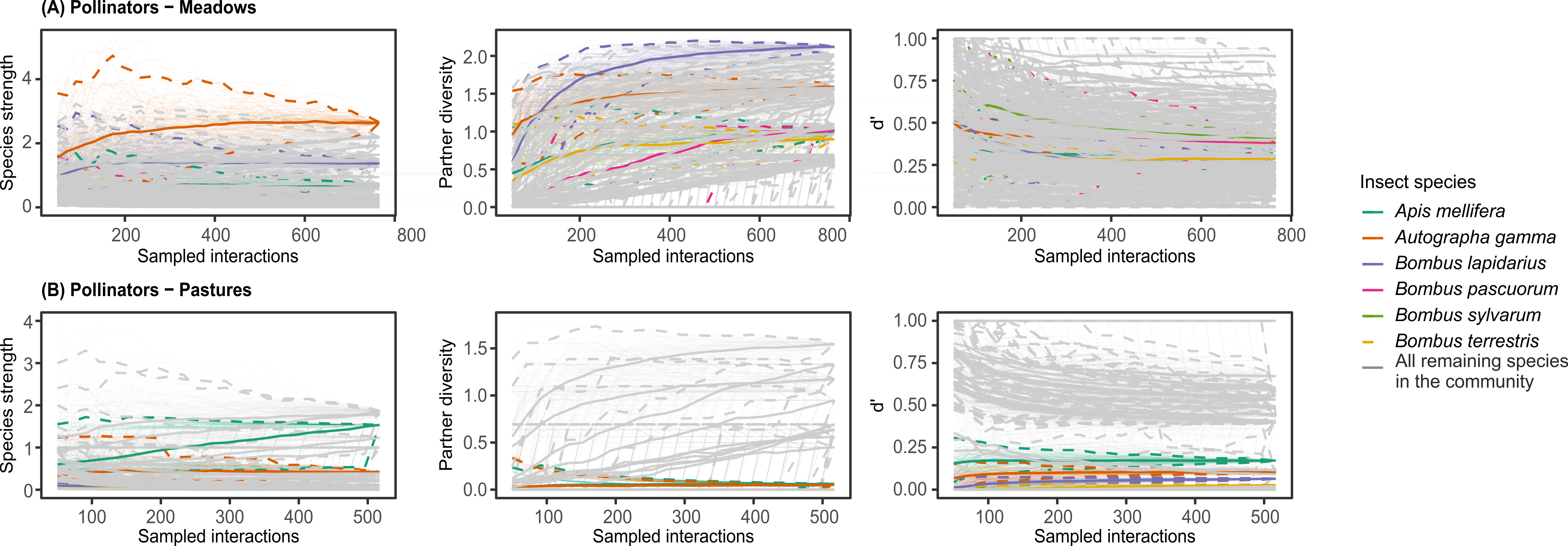

Supplement: S6 Fig — Species level metrics of A. mellifera, A. gamma and B. lapidarius in comparison to all other pollinator species in the networks of: (A) hay meadows and (B) pastures. Metrics have been rarefied for pooled interactions. Shaded areas represent 95% confidence intervals. Besides A. mellifera, A. gamma and B. lapidarius which were the most frequent pollinators of T. repens, we highlighted also other Bombus species frequently observed on the flowers for reference. (TIF) [file pone.0263576.s009.tif]
