# Supplementary material for: Intensive grazing alters the diversity, composition and structure of plant-pollinator interaction networks in Central European grasslands
Source: PLoS One. 2022 Mar 11;17(3):e0263576. doi: 10.1371/journal.pone.0263576 (PMC8916670; doi:10.1371/journal.pone.0263576)
Supplement: S4 Fig — Module structure of (A) hay meadows and (B) pastures illustrating the taxonomic and functional composition of each module. Grey shaded squares indicate weighted interactions (darker colours indicate higher interaction frequency). Colour codes indicate the functional group for each species and these colours follow those in Fig 3 (grey denotes species whose functional traits could not be assessed, most of which were Diptera). Bold names indicate the 10 most abundant plant and pollinator species. (ZIP) [file pone.0263576.s007.zip › S4a_Fig.pdf]

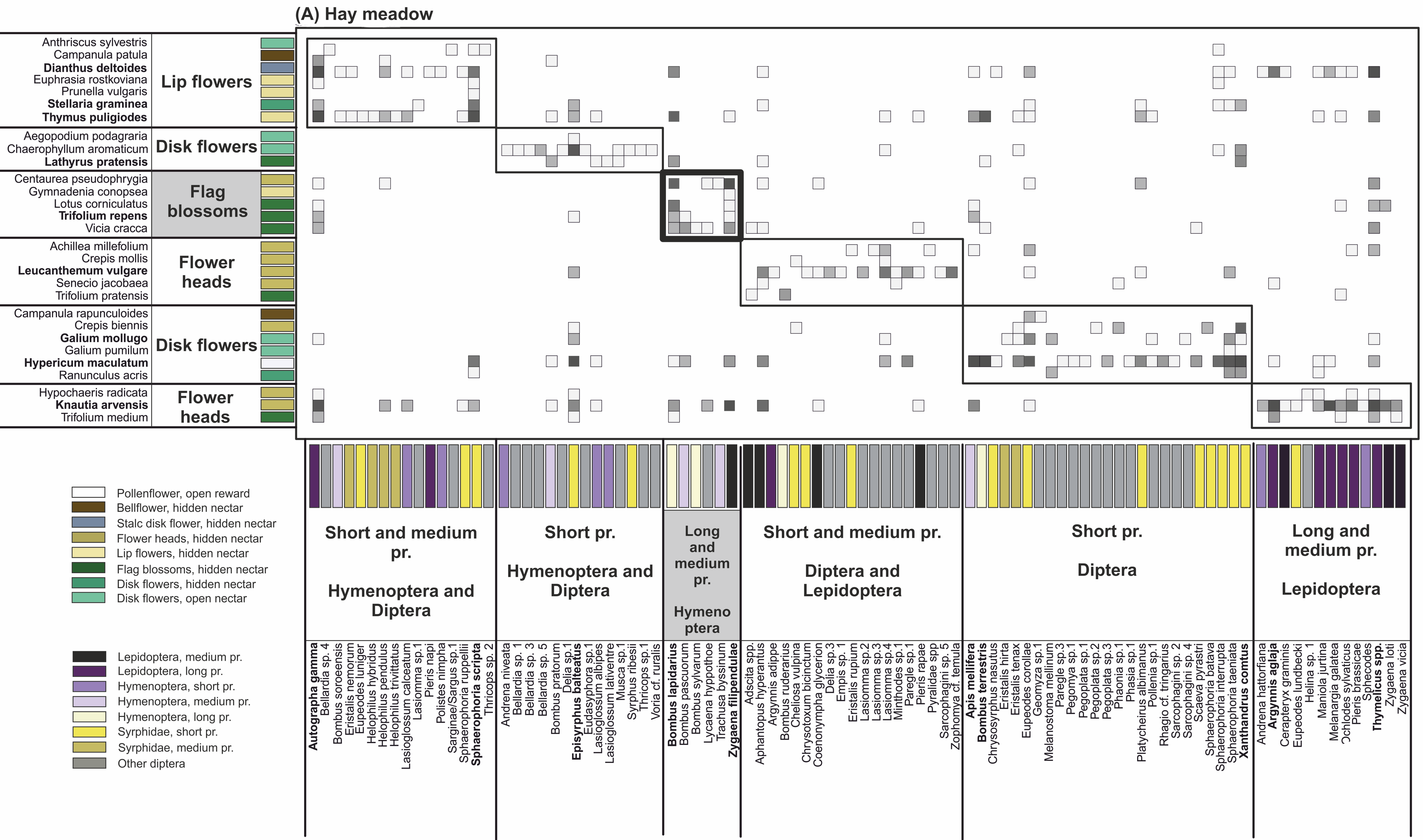

(A) Hay meadow
Anthriscus sylvestris
Campanula patula
Dianthus deltoides
Euphrasia rostkoviana
Prunella vulgaris
Stellaria graminea
Thymus puligiodes
Lip flowers
Aegopodium podagraria
Chaerophyllum aromaticum
Lathyrus pratensis
Disk flowers
Centaurea pseudophrygia
Gymnadenia conopsea
Lotus corniculatus
Trifolium repens
Vicia cracca
Flag blossoms
Achillea millefolium
Crepis mollis
Leucanthemum vulgare
Senecio jacobaea
Trifolium pratensis
Flower heads
Campanula rapunculoides
Crepis biennis
Galium mollugo
Galium pumilum
Hypericum maculatum
Ranunculus acris
Disk flowers
Hypochaeris radicata
Knautia arvensis
Trifolium medium
Flower heads
Pollenflower, open reward
Bellflower, hidden nectar
Stalc disk flower, hidden nectar
Flower heads, hidden nectar
Lip flowers, hidden nectar
Flag blossoms, hidden nectar
Disk flowers, hidden nectar
Disk flowers, open nectar
Lepidoptera, medium pr.
Lepidoptera, long pr.
Hymenoptera, short pr.
Hymenoptera, medium pr.
Hymenoptera, long pr.
Syrphidae, short pr.
Syrphidae, medium pr.
Other diptera
Short and medium pr.
Hymenoptera and Diptera
Short pr.
Hymenoptera and Diptera
Long and medium pr.
Hymeno ptera
Short and medium pr.
Diptera and Lepidoptera
Short pr.
Diptera
Long and medium pr.
Lepidoptera
Autographa gamma
Bellardia sp. 4
Bombus soroeensis
Eristalis nemorum
Eupeodes luniger
Helophilus hybridus
Helophilus pendulus
Helophilus trivittatus
Lasioglossum calceatum
Lasiomma sp.1
Pieris napi
Polistes nimpha
Sarginae/Sargus sp.1
Sphaerophoria ruppellii
Sphaerophoria scripta
Thricops sp. 2
Andrena niveata
Bellardia sp. 1
Bellardia sp. 3
Bellardia sp. 5
Bombus pratorum
Delia sp.1
Episyrphus balteatus
Eudasyphora sp.1
Lasioglossum albipes
Lasioglossum lativentre
Musca sp.1
Syrphus ribesii
Thricops sp.1
Voria cf. ruralis
Bombus lapidarius
Bombus pascuorum
Bombus sylvarum
Lycaena hyppothoe
Trachusa byssinum
Zygaena filipendulae
Adscita spp.
Aphantopus hyperantus
Argynnis adippe
Bombus ruderarius
Cheilosa vulpina
Chrysotoxum bicinctum
Coenonympha glycerion
Delia sp.3
Empis sp. 1
Eristalis rupium
Lasiomma sp.2
Lasiomma sp.3
Lasiomma sp.4
Minthodes sp.1
Paregle sp.1
Pieris rapae
Pyralidae spp
Sarcophagini sp. 5
Zophomya cf. temula
Apis mellifera
Bombus terrestris
Chrysosyrphus nasutus
Eristalis hirta
Eristalis tenax
Eupeodes corollae
Geomyza sp.1
Melanostoma mellinum
Paregle sp.3
Pegomya sp.1
Pegoplata sp.1
Pegoplata sp.2
Pegoplata sp.3
Phaonia sp.1
Phasia sp.1
Platycheirus albimanus
Pollenia sp.1
Rhagio cf. tringarius
Sarcophagini sp. 2
Sarcophagini sp. 4
Scaeva pyrastri
Sphaerophoria batava
Sphaerophoria interrupta
Sphaerophoria taeniata
Xanthandrus comtus
Andrena hattorfiana
Argynnis aglaja
Cerapteryx graminis
Eupeodes lundbecki
Helina sp. 1
Maniola jurtina
Melanargia galatea
Ochlodes sylvaticus
Pieris brassicae
Sphecodes
Thymelicus spp.
Zygaena loti
Zygaena vicia
