# Supplementary material for: Intensive grazing alters the diversity, composition and structure of plant-pollinator interaction networks in Central European grasslands
Source: PLoS One. 2022 Mar 11;17(3):e0263576. doi: 10.1371/journal.pone.0263576 (PMC8916670; doi:10.1371/journal.pone.0263576)
Supplement: S4 Fig — Module structure of (A) hay meadows and (B) pastures illustrating the taxonomic and functional composition of each module. Grey shaded squares indicate weighted interactions (darker colours indicate higher interaction frequency). Colour codes indicate the functional group for each species and these colours follow those in Fig 3 (grey denotes species whose functional traits could not be assessed, most of which were Diptera). Bold names indicate the 10 most abundant plant and pollinator species. (ZIP) [file pone.0263576.s007.zip › S4b_Fig.pdf]

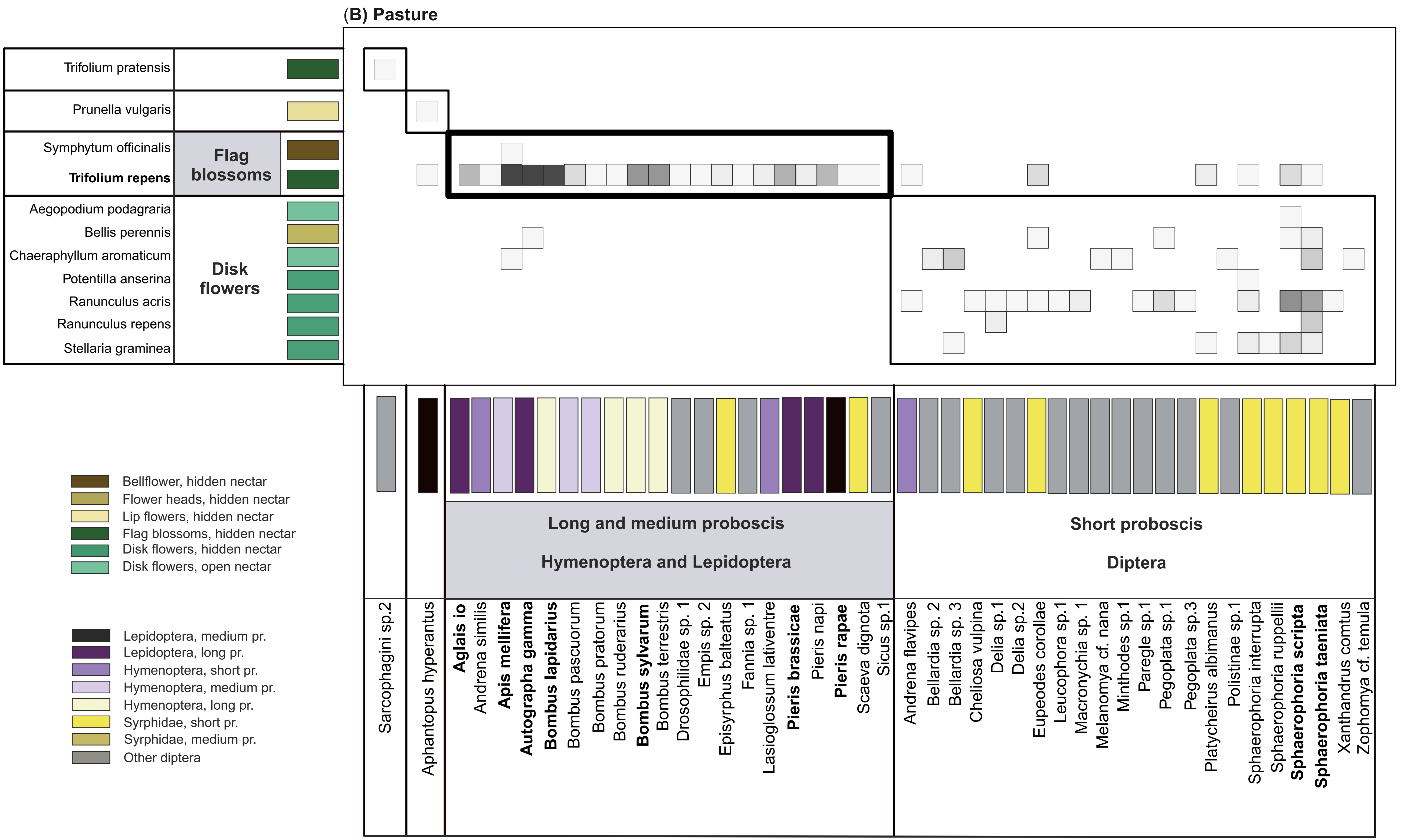
(B) Pasture
Trifolium pratensis
Prunella vulgaris
Symphytum officinalis
Trifolium repens
Flag blossoms
Aegopodium podagraria
Bellis perennis
Chaeraphyllum aromaticum
Potentilla anserina
Ranunculus acris
Ranunculus repens
Stellaria graminea
Disk flowers
Bellflower, hidden nectar
Flower heads, hidden nectar
Lip flowers, hidden nectar
Flag blossoms, hidden nectar
Disk flowers, hidden nectar
Disk flowers, open nectar
Lepidoptera, medium pr.
Lepidoptera, long pr.
Hymenoptera, short pr.
Hymenoptera, medium pr.
Hymenoptera, long pr.
Syrphidae, short pr.
Syrphidae, medium pr.
Other diptera
Long and medium proboscis
Hymenoptera and Lepidoptera
Short proboscis
Diptera
Sarcophagini sp.2
Aphantopus hyperantus
Aglais io
Andrena similis
Apis mellifera
Autographa gamma
Bombus lapidarius
Bombus pascuorum
Bombus pratorum
Bombus ruderarius
Bombus sylvarum
Bombus terrestris
Drosophilidae sp. 1
Empis sp. 2
Episyrphus balteatus
Fannia sp. 1
Lasioglossum lativentre
Pieris brassicae
Pieris napi
Pieris rapae
Scaeva dignota
Sicus sp.1
Andrena flavipes
Bellardia sp. 2
Bellardia sp. 3
Cheliosa vulpina
Delia sp.1
Delia sp.2
Eupeodes corollae
Leucophora sp.1
Macronychia sp. 1
Melanomya cf. nana
Minthodes sp.1
Paregle sp.1
Pegoplata sp.1
Pegoplata sp.3
Platycheirus albimanus
Polistinae sp.1
Sphaerophoria interrupta
Sphaerophoria ruppellii
Sphaerophoria scripta
Sphaerophoria taeniata
Xanthandrus comtus
Zophomya cf. temula
