# Supplementary material for: Intensive grazing alters the diversity, composition and structure of plant-pollinator interaction networks in Central European grasslands
Source: PLoS One. 2022 Mar 11;17(3):e0263576. doi: 10.1371/journal.pone.0263576 (PMC8916670; doi:10.1371/journal.pone.0263576)
Supplement: S1 Appendix — (DOCX) [file pone.0263576.s010.docx]

**S1 Appendix. Indirect measure of land-use intensity***.*

Using the composition of plants with different ecological tolerances we tested whether the analysed hay meadows and pastures differed significantly in their management intensity. Specifically, we compared the ecological indicator values for grazing-, trampling- and nutrient-tolerance of the plant species occurring within sites belonging to the two management types (ecological indicator values, summarized in S2 Table, were derived from: [*http://statedv.boku.ac.at/zeigerwerte*](http://statedv.boku.ac.at/zeigerwerte) and [*www.biolflor.de*](http://www.biolflor.de)*,* [1,2]). Increased frequency of species with high tolerance levels for grazing, trampling and nutrient input should be indicative for intensively managed sites [3]. We tested whether the analysed pastures differed significantly from hay meadows in the intensity of disturbance (grazing and trampling) and nutrient input by relying on a non-parametric multivariate ANOVA-type test in the R package “npmv”. This analysis is appropriate for comparisons with small sample sizes and ordinal variables [4]. The function “nonpartest”, which performs a one-way non-parametric (F-approximations for ANOVA Type tests) multivariate analysis with 1000 permutations, was used to assess the overall difference in the distribution of plant species with various levels of tolerance for trampling, grazing and nutrient input in pastures and hay meadows.

Our indirect assessment of land-use intensity showed that plant species tolerant of high degrees of trampling, grazing and nitrogen input were more frequent in pastures than in hay meadows (non-parametric ANOVA type test, F = 3.28; p = 0.029). This confirmed our assessment of higher management intensity in pastures, in comparison to hay meadows.

**References**

1. Ellenberg HH. Vegetation Ecology of Central Europe. Cambridge University Press; 1988.

2. Klotz S, Kühn I, Durka W. BIOLFLOR - Eine Datenbank zu biologisch-ökologischen Merkmalen der Gefäßpflanzen in Deutschland. Schriftenreihe für Vegetationskunde. 38th ed. 2002.

3. Ellenberg HH, Weber HE, Düll E, Wirth V, Werner W, Paulissen D. Zeigerwerte von Pflanzen in Mitteleuropa. 2nd ed. Göttingen: Erich Goltze KG; 1992.

4. Burchett W, Ellis A. npmv: Nonparametric Comparison of Multivariate Samples. 2017.
